# Soluble flagellin coimmunization attenuates Th1 priming to Salmonella and clearance by modulating dendritic cell activation and cytokine production

**DOI:** 10.1002/eji.201545564

**Published:** 2015-06-24

**Authors:** Adriana Flores‐Langarica, Saeeda Bobat, Jennifer L. Marshall, Juan Carlos Yam‐Puc, Charlotte N. Cook, Karine Serre, Robert A. Kingsley, Leopoldo Flores‐Romo, Satoshi Uematsu, Shizuo Akira, Ian R. Henderson, Kai M. Toellner, Adam F. Cunningham

**Affiliations:** ^1^ Division of Immunity and InfectionInstitute of Biomedical ResearchUniversity of BirminghamBirminghamUK; ^2^ Deparamento de Biologia Celular. CINVESTAV. MexicoD.F. Mexico; ^3^ Instituto de Medicina MolecularFaculdade de Medicina, Universidade de LisboaLisbonPortugal; ^4^ The Institute of Food ResearchNorwich Research ParkNorwichUK; ^5^ International Research and Development Centre for Mucosal VaccineInstitute for Medical ScienceThe University of TokyoTokyoJapan; ^6^ Laboratory of Host DefenseWorld Premier International Immunology Frontier Research CenterOsaka UniversitySuita OsakaJapan; ^7^ Department of Host DefenseResearch Institute for Microbial DiseasesOsaka UniversitySuita OsakaJapan

**Keywords:** Dendritic cell activation, Flagellin, Priming, *Salmonella* Typhimurium, Th1 cells

## Abstract

Soluble flagellin (sFliC) from *Salmonella* Typhimurium (STm) can induce a Th2 response to itself and coadministered antigens through ligation of TLR5. These properties suggest that sFliC could potentially modulate responses to Th1 antigens like live STm if both antigens are given concurrently. After coimmunization of mice with sFliC and STm there was a reduction in Th1 T cells (T‐bet^+^IFN‐γ^+^ CD4 T cells) compared to STm alone and there was impaired clearance of STm. In contrast, there was no significant defect in the early extrafollicular B‐cell response to STm. These effects are dependent upon TLR5 and flagellin expression by STm. The mechanism for these effects is not related to IL‐4 induced to sFliC but rather to the effects of sFliC coimmunization on DCs. After coimmunization with STm and sFliC, splenic DCs had a lower expression of costimulatory molecules and profoundly altered kinetics of IL‐12 and TNFα expression. Ex vivo experiments using in vivo conditioned DCs confirmed the effects of sFliC were due to altered DC function during a critical window in the coordinated interplay between DCs and naïve T cells. This has marked implications for understanding how limits in Th1 priming can be achieved during infection‐induced, Th1‐mediated inflammation.

## Introduction

DCs can efficiently capture, process, and present antigen to T cells in the T zones of secondary lymphoid tissues such as the spleen. If cognate interactions between these two cell types results in T‐cell priming, then cells can differentiate to become Th cells 1. In vivo, the direction of Th‐cell differentiation is influenced by the nature of the antigen. Thus, Th1 responses are induced by intracellular bacteria such as *Salmonella enterica* serovar Typhimurium (STm), Th17 responses are characteristic of pneumococcal infection and Th2 responses are observed after exposure to antigens such as helminths and alum‐precipitated proteins such as OVA. Regulating the direction and magnitude of the Th response is important since inappropriate responses are associated with a failure to control infection or enhanced pathology and inflammation 2, 3. For instance, T‐bet‐deficient mice generate T‐cell responses to STm but fail to clear the bacteria due to an impairment in Th1 development 2, 4, 5, 6, 7.

To understand how the extent of the Th response is regulated it is necessary to appreciate the factors that drive the Th response down one pathway or another. One important element is the environment in which the antigen is encountered by the immune system 4, 8. Thus, OVA‐specific OT‐II CD4 T cells responding to alum‐precipitated OVA polarize to Th2, but when the same antigen is expressed within an attenuated strain of STm then a Th1 response is generated 4, 9. In addition, infectious history can selectively influence the T‐cell response. For instance, during coinfection with STm and the helminth *Nippostrongylus brasiliensis* there is a diminished Th2 response to the helminth, whereas the Th1 response to STm remains largely unaffected 10. One possible interpretation of this is that Th1 responses are more resistant to modulation than other types of responses.

To better understand the principles behind the regulation of Th‐cell responses some groups, including ourselves, have compared the T‐cell response induced to the same antigen when given in purified form or in its native context as part of a live bacterium. One molecule that is helpful for this is flagellin, which is the component antigen of the flagellar filament 11. This protein is exposed on the bacterial surface, so it is available to B cells, can be expressed at high levels and is a significant target of the T‐cell response to STm 12. Furthermore, when administered in purified form, soluble flagellin (sFliC), from STm has the valuable property of having auto‐adjuvant activity through its ligation of TLR5 and other mechanisms 13, 14, 15. Therefore responses to this protein can be assessed in the absence of potentially biasing influences such as exogenous adjuvant. Previous studies have shown that in the spleen the sFliC‐specific response is Th1 when it is encountered in its native context as part of STm, with a robust induction of T‐bet and type‐specific cytokine IFN‐γ. In contrast, after immunization with sFliC there is a clear induction of Th2 features such as GATA‐3 mRNA and IL‐4 protein. The dichotomy of the T‐cell response is also reflected in the B‐cell response to sFliC, with the direction of IgG switching to live bacteria primarily to a Th1‐reflecting IgG2a, whereas to sFliC it is a Th2‐reflecting IgG1 4, 16.

Since the response to the same antigen can differ depending upon the context in which it is encountered, it suggested to us that DCs were important, since they are at the center of directing Th differentiation 17. Previously, our studies have shown a major role for monocyte‐derived DCs (moDCs) in Th1 priming through their capacity to collaborate with conventional DCs (cDCs) 18. This shows that factors influencing the biology of DCs have a corresponding effect on their capacity to prime Th responses. Therefore we asked what the consequence for the host T‐cell response to STm would be after coadministration of live STm and sFliC. These studies showed that the presence of sFliC reduced the numbers of Th1 cells after STm compared to STm alone via its TLR5‐dependent effects on DCs. Therefore, sFliC can modify the response to STm through modulating DC function.

## Results

### Mice coimmunized with STm and sFliC have lower IFN‐γ T‐cell responses

**Figure 1:**
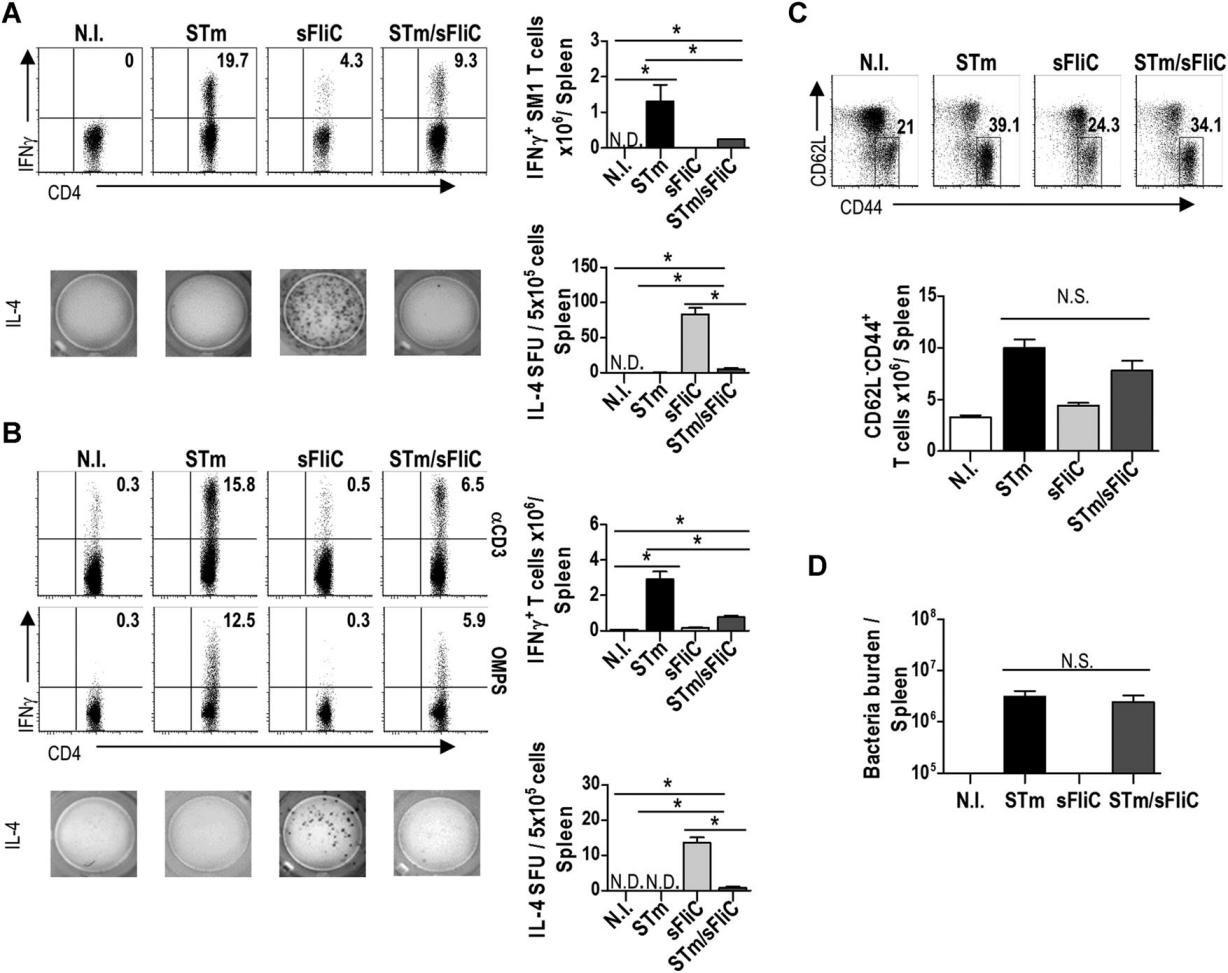
sFliC coimmunization with STm influences the T‐cell IFN‐γ response. (A) SM1 splenocytes were adoptively transferred into TCRβδ^−/−^ mice; 24 h posttransfer (A) chimeras and (B) WT mice were immunized with PBS (white) or 5 × 10^5^ STm (black), 20 μg sFliC (light gray) or both (dark gray). IFN‐γ levels in CD4^+^ T cells (CD3^+^CD4^+^) 7 days postimmunization after in vitro restimulation with (A) aCD3 or (B) aCD3 or OMPS were evaluated by FACS. Representative FACS plots and absolute numbers of intracellular IFN‐γ in (A) SM1 CD4^+^ T cells and (B) CD4^+^ T cells are shown; numbers in the quadrant represent quadrant frequency of the shown plot. (A and B, bottom right) Quantification of the IL‐4 SFUs/5 × 10^5^ cells by ELISPOT. Data are shown as mean ± SD (*n* = 3 mice/group) and are representative of three independent experiments. *p ≤ 0.01, by 1 way ANOVA. (C) Representative FACS plots and quantification of the frequency of CD44^+^CD62L^−^CD4^+^ T cells at day 7. Data are shown as mean ± SD (*n* = 4 mice/group) and are representative of three independent experiments. *p ≤ 0.01, by 1 way ANOVA. (D) Bacteria burden in the spleen of mice immunized with STm, FliC, or both STm/sFliC at day 7. Data are shown as mean ± SD (*n* = 3 mice/group) and are representative of three independent experiments.

We have previously shown that sFliC induces a Th2‐dominated response characterized by a marked induction of IL‐4 and GATA‐3 mRNA expression in antigen‐specific T cells, but not IFN‐γ 4, 19. In contrast, when FliC is encountered as part of STm a Th1 response is induced, characterized by IFN‐γ and T‐bet expression. This led us to examine if there was any modulation of the response if the immune system encountered both antigens simultaneously. To do this, SM1 transgenic T cells (specific for a peptide in STm flagellin) were used to generate chimeras that were then immunized with PBS, STm, sFliC, or STm and sFliC. As expected and shown in Fig. 1A, sFliC induced little IFN‐γ in SM1 T cells while STm induced a robust IFN‐γ response 4. However, in mice that received both antigens there was a reduction in the proportion and number of IFN‐γ‐producing SM1 T cells in comparison with the STm only infected group. In contrast, IL‐4 production was readily detected in sFliC‐immunized mice by ELISPOT, but not in the other groups. This included mice that received STm and sFliC together (Fig. 1A and 4).

To test whether sFliC and STm coimmunization could similarly impact the endogenous CD4 T‐cell (gating strategy shown in Supporting Information Fig. 1A) response, WT mice were immunized with STm, sFliC, or both. After 7 days a diminution of the IFN‐γ response was observed in the mice coimmunized with STm and sFliC as seen in the transgenic SM1 system. This response was not only observed in anti‐CD3 restimulated cultures but also after restimulation of antigen‐specific T cells with purified STm outer membrane proteins (OMPS) (Fig. 1B). IL‐4 was detected in the sFliC primed mice in the absence of transgenic T cells. These changes in cytokine responses were not due to fewer activated T cells since the proportion and the absolute numbers of CD62L^lo^CD44^+^ CD4 T cells were similar at day 7 in both groups that received STm (Fig. 1C). We tested if this modulation of the T‐cell response was associated with an altered frequency or number of Treg cells or if the effect was IL‐10 mediated. At day 7 postimmunization, when we observed the diminution of IFN‐γ expression by T cells there was no difference in the numbers of Treg cells between the mice immunized with STm or STm/sFliC (Supporting Information Fig. 2A), nor was there a difference between WT and IL‐10‐deficient mice (anti‐CD3 restimulation shown in Supporting Information Fig. 2B and similar results were observed after restimulation with OMPS).

To address if this diminution of the IFN‐γ response impacted on bacterial clearance at this time the bacterial burden between the groups immunized with STm or STm/sFliC was assessed, which showed no significant difference (Fig. 1D). Thus coimmunization of sFliC and STm alters the IFN‐γ response to STm and the IL‐4 response to sFliC in both transgenic and endogenous CD4 T cells without altering the capacity of mice to control the early stages infection.

**Figure 2:**
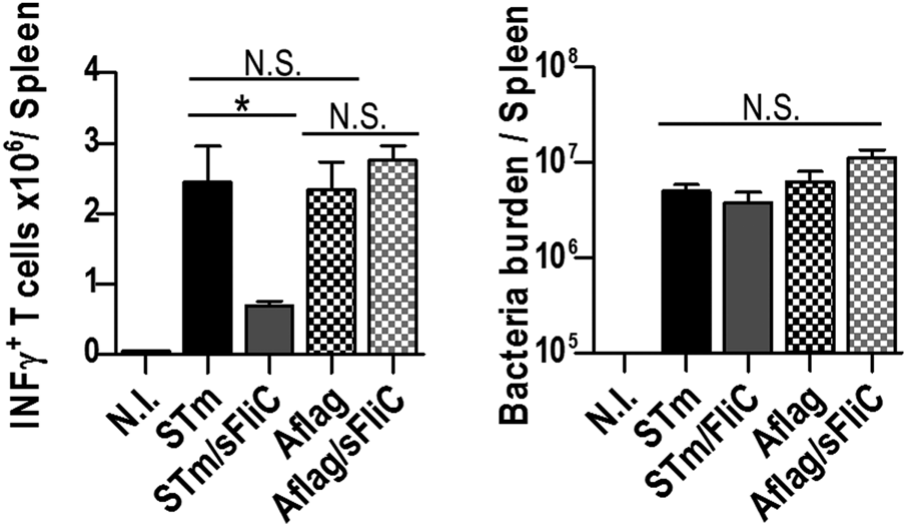
Flagellin expression on the bacteria is required for IFN‐γ modulation. (A) WT mice were non‐immunized or immunized with 5 × 10^5^ STm, STm/sFliC (20 μg), 5 × 10^5^ Aflagellated STm or Aflagellated STm/sFliC (20 μg). Intracellular IFN‐γ in CD4^+^ T cells was evaluated 7 days postimmunization after in vitro restimulation with aCD3. (Left) Absolute numbers of IFN‐γ^+^ CD4^+^ T cells and (right) splenic bacteria burden was measured by direct culture. Data are shown as mean ± SD (*n* = 3 mice/group) and are representative of three independent experiments. *p ≤ 0.01, by 1 way ANOVA.

We then examined if these effects require the expression of flagellin on the bacterium. To test this possibility, WT mice were immunized as before alongside groups of mice infected with aflagellate STm or aflagellate STm with sFliC. Flagellated and aflagellate STm induced similar numbers of IFN‐γ^+^ T cells. Unexpectedly, no reduction in the number of IFN‐γ^+^ T cells was observed when sFliC was coimmunized with aflagellated bacteria. This was not due to a difference in the bacterial burden since these were similar between the groups (Fig. 2). This was also not due to differences in immune responses between these strains, since at early time points no differences were observed in cDC and moDC numbers and at day 7 equivalent numbers of activated T cells and similar Ab responses were observed (Supporting Information Fig. 3). Therefore, the capacity of sFliC to impair Th1 responses is dependent upon parallel flagellin expression by the bacterium.

### STm and sFliC coimmunization impairs T‐bet^+^ T‐cell numbers in a TLR5‐dependent manner

**Figure 3:**
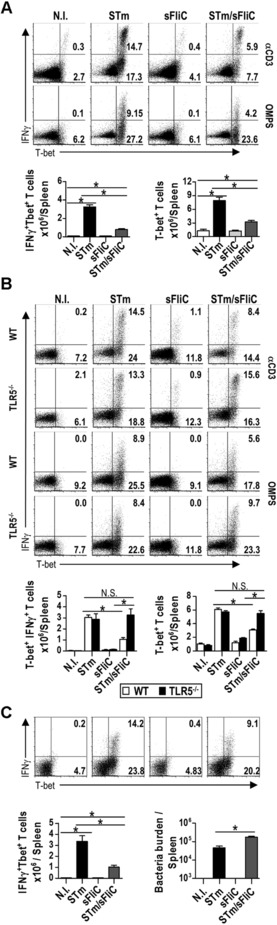
sFliC influences the Th1 response to STm in a TLR5‐dependent manner. (A) WT mice were nonimmunized (white) or immunized with 5 × 10^5^ STm (black), 20 μg sFliC (light gray), or both (dark gray). (Top) Representative FACS plots show intracellular IFN‐γ and T‐bet expression on T cells (CD3^+^CD4^+^) 7 days postimmunization after in vitro restimulation (αCD3 or OMPS). (Bottom) Absolute number of IFN‐γ^+^T‐bet^+^ T cells and T‐bet^+^ T cells per spleen were measured by FACS. (B) WT (white) or TLR5^−/−^ mice (black) were immunized as in A, T‐bet expression and intracellular IFN‐γ after in vitro restimulation (αCD3 and OMPS) were evaluated by FACS, 7 days postinfection. (Top) Representative plots show intracellular IFN‐γ and T‐bet expression on T cells (CD3^+^CD4^+^). (Bottom) Absolute number of T‐bet^+^IFN‐γ^+^ T cells and of T‐bet^+^ T cells per spleen (C) WT mice were nonimmunized (white) or immunized with STm (black), sFliC (light gray) or both (dark gray). (Top) Representative FACS plots show intracellular IFN‐γ and T‐bet expression on T cells (CD3^+^CD4^+^) 21 days postimmunization after in vitro restimulation (αCD3). (Bottom) Absolute number of IFN‐γ^+^Tbet^+^ T cells and bacteria burden were measured by FACS. (A–C) Data are representative of three independent experiments. Data in graphs are shown as mean ± SD (*n* = 4 mice/group) *p ≤ 0.01, by 1 way ANOVA.

IFN‐γ‐production by T cells after STm infection is dependent upon the transcription factor T‐bet 6. To assess at what stage during Th1 differentiation, sFliC was modulating the IFN‐γ production we examined whether coimmunization of STm and sFliC simply restricted the numbers of IFN‐γ producing T cells, or whether its impact was more profound, and impaired the induction of T‐bet in T cells. In comparison with the STm‐immunized group, the coimmunized mice had fewer IFN‐γ^+^T‐bet^+^ CD4 T cells and fewer numbers of total T‐bet^+^ CD4 T cells and even polyfunctional IFN‐γ^+^TNFα^+^ CD4 T cells. Notably, all IFN‐γ^+^ T cells also coexpressed T‐bet, independently of whether the cells were restimulated with anti‐CD3 or OMPS (Fig. 3A and data not shown). We hypothesized that since TLR5 is the extracellular receptor for sFliC, that this receptor may play a central role in this modulation of Th1 differentiation. To test this we assessed Th1 cell numbers in coimmunized WT and TLR5^−/‐^ mice. Numbers of T‐bet^+^ T cells were similar in WT and TLR5^−/−^ mice given STm alone or sFliC alone. In agreement with our hypothesis, the coimmunized WT group had fewer IFN‐γ^+^T‐bet^+^ T cells and T‐bet^+^ T cells whereas in the TLR5^−/−^ group there was no diminution in the proportion or numbers of T cells of either phenotype (Fig. 3B). At this day 7 time‐point control of infection is independent of T cells 4, 7, so to examine the impact of coimmunization on the later T‐cell response, bacterial burdens were examined at 3 weeks after infection, when T‐cell help is well established. Lower numbers of T‐bet^+^IFN‐γ^+^ CD4^+^ T cells were detected at day 21 and coimmunized mice had higher bacterial burdens at this time (Fig. 3C). Therefore, sFliC coimmunization impairs the Th1 programme at the level of T‐bet induction and this occurs in a TLR5‐dependent manner. This effect persists and is reflected in a diminished rate of clearance of bacteria from the spleen.

### sFliC coimmunization does not modify the T‐independent early B‐cell response to STm

**Figure 4:**
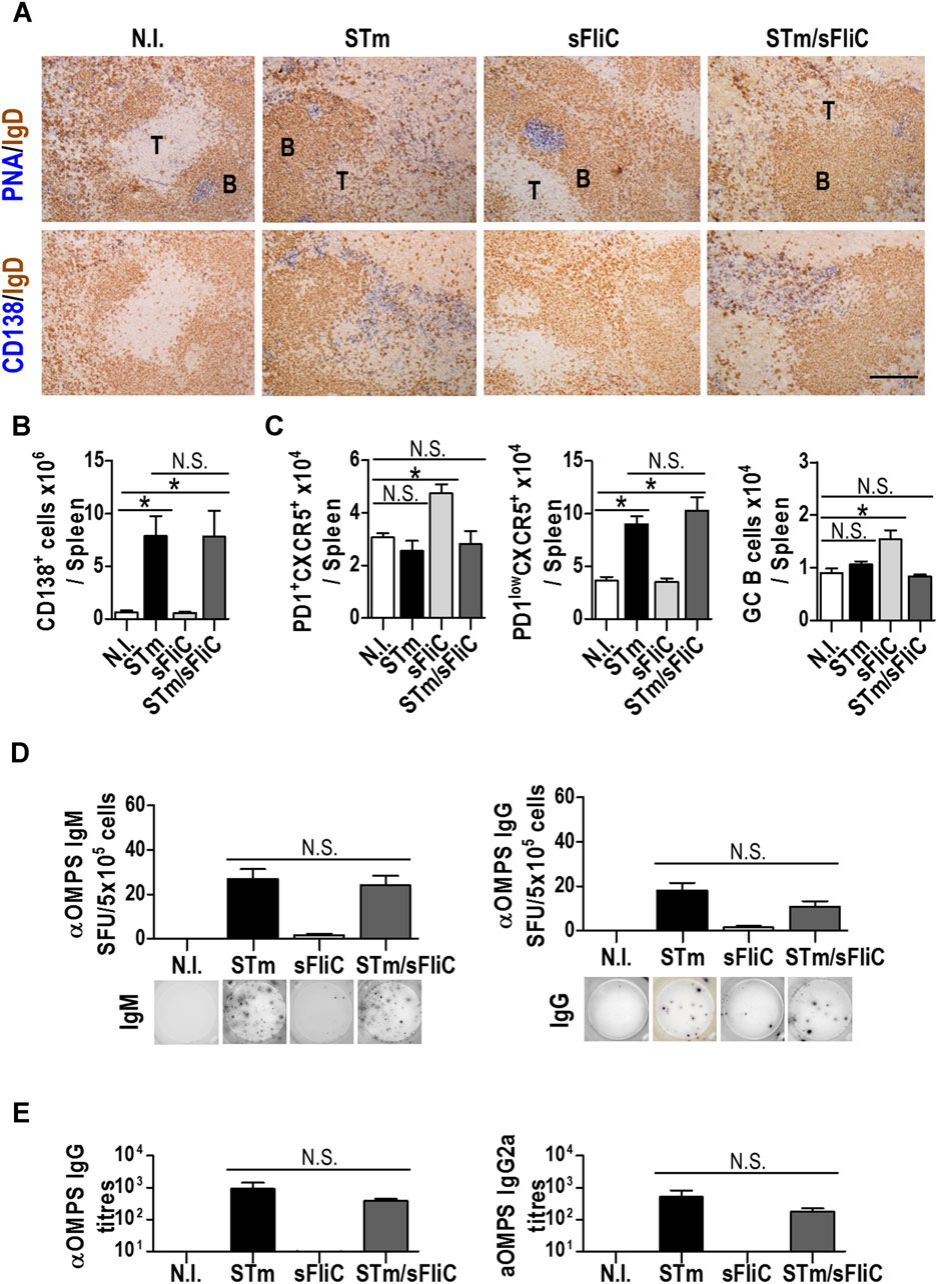
sFliC coimmunization with STm does not affect the T‐independent B cell response to STm. WT mice were nonimmunized (white) or immunized with 5 × 10^5^ STm (black), 20 μg sFliC (light gray) or both (dark gray) for 7 days. (A) Immunohistological assessment of the splenic GCs (PNA, blue; IgD, brown) and plasma cells (CD138, blue; IgD, brown). Representative photomicrographs of three independent experiments are shown. T; T zone, B; B zone, scale bar: 200 μm. Quantification of (B) CD138^+^ B cells and of (C) Tfh cells (PD1^+^CXCR5^+^), PD1^low^CXCR5^+^ T cells, and GC B cells (Fas^+^GL7^+^) in the spleen was made by FACS. (Gating strategies shown in Supporting Information Fig. 1B). (D) IgM and IgG response to STm OMPS 7 days postimmunization measured by ELISPOT. (E) Anti‐OMPS serum IgG and IgG2a titers were evaluated 21 days postimmunization by ELISA. (B–E) Data are shown as mean ± SD (*n* = 4 mice/group) and are representative of three independent experiments. *p ≤ 0.01, by 1 way ANOVA.

Coimmunization with sFliC had a dramatic impact on the induction of Th1 cells, but it was also possible this may influence B‐cell responses too. In this model, the B‐cell response to STm is atypical since there is an extensive and rapid extrafollicular response that occurs in the absence of germinal centers and the generation of high‐affinity antibody to the bacterium 20, 21. The induction of the B‐cell response is T‐independent but switching requires T cells. Coimmunization with sFliC did not alter the induction of extrafollicular plasma cells and plasmablasts (Fig. 4A and B). Nor did it alter numbers of T cells (PD1^lo^CXCR5^+^) with a phenotype associated with extrafollicular switching after Salmonella infection and that are diminished in the absence of the transcription factor BCL6 22. Nevertheless, there were fewer cells with features of germinal center‐associated T‐follicular helper cells (Tfh, PD1^+^CXCR5^+^) and germinal center B cells (Fas^+^GL7^+^) compared to mice that only received sFliC (Fig. 4C, gating strategy shown in Supporting Information Fig. 1B). We also addressed the antigen specificity of the response by ELISPOT. IgM and IgG specific cells to OMPS were detected with similar frequencies in mice immunized with STm or coimmunized with STm/sFliC (Fig. 4D). Finally, we evaluated anti‐OMPS Ab titers by ELISA 21 days postimmunization and confirmed that there was no significant difference between the groups immunized with STm or coimmunized with STm/sFliC (Fig. 4E). Therefore, coimmunization with sFliC does not impair the induction of B‐cell responses to STm.

### Coimmunization with sFliC impairs the early activation of T cells

**Figure 5:**
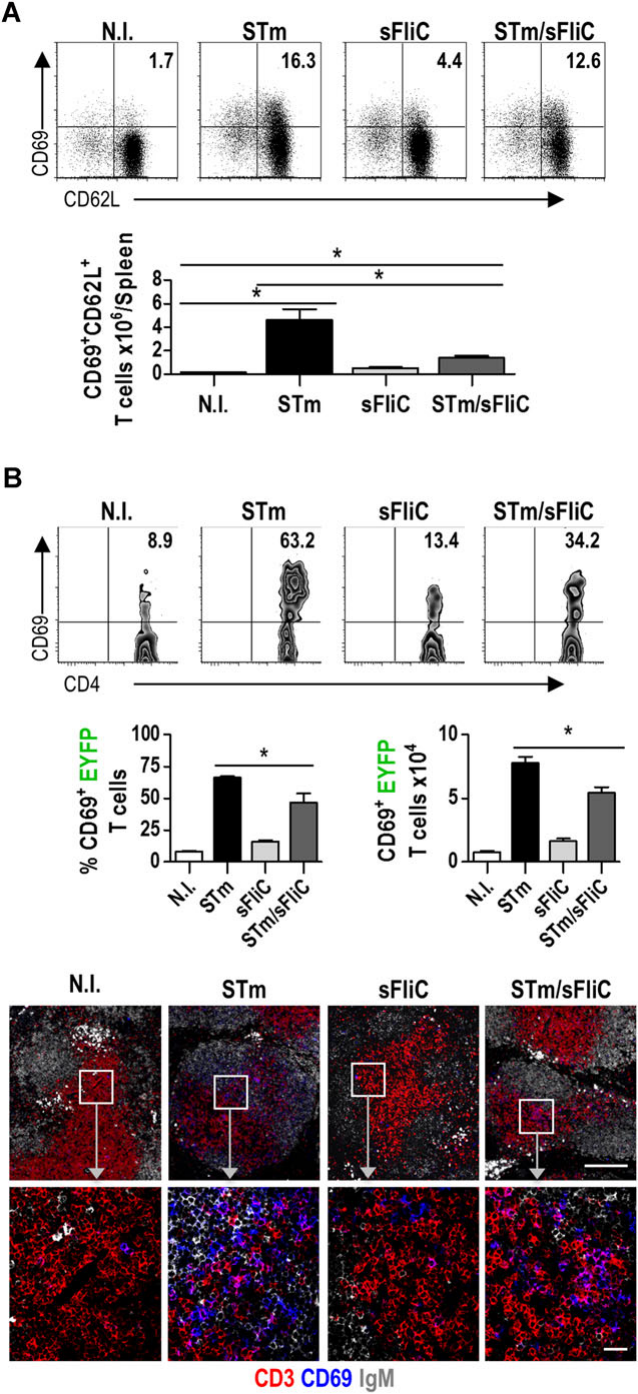
Coimmunization of sFliC and STm leads to a defective early T‐cell activation in comparison with STm immunization alone. (A) WT mice were nonimmunized (white) or immunized with 5 × 10^5^ STm (black), 20 μg sFliC (light gray), or both (light gray). Early T‐cell activation was evaluated 24 h postimmunization. (Top) Representative FACS plots show T‐cell (CD3^+^CD4^+^CD44^−^) activation by the expression of CD69 and CD62L; numbers represent frequency within the quadrant. (Bottom) Quantification of CD69^+^CD62L^+^ T cells at that time point. (B) Early T‐cell activation was evaluated in a chimeric system using STm antigen‐experienced T cells. EYFP mice were STm infected (5 × 10^5^ SL3261) for 7 days. T cells were isolated and MACS‐enriched to transfer 10^7^ EYFP T cells into WT mice, 24 h posttransfer mice were nonimmunized or immunized with 5 × 10^5^ STm (black), 20 μg sFliC (light gray), or both (dark gray), activation was evaluated on EYFP^+^ T cells. (Top) Representative plots show the expression of CD69 on EYFP^+^TCRαβ^+^CD4^+^ cells (middle) frequency and the absolute number of CD69^+^EYFP^+^ T cells at 24 h postimmunization. (Bottom) Composite confocal representative images of spleens from WT mice 24 h postimmunization. Images show CD69 (blue), CD3 (red), and IgM (white). (Top) Low magnification (scale bar: 200 μm) and (bottom) higher magnification of the boxed areas (scale bar: 20 μm) are shown. (A and B) Data are shown as mean ± SD (*n* = 3‐4 mice/group) and are representative of three independent experiments. *p ≤ 0.01, by 1 way ANOVA.

We then tried to pinpoint how early in the response sFliC coimmunization had this effect. Assessment of early T‐cell activation, by examining the expression of CD69 and CD62L (in CD44^−^CD3^+^CD4^+^ T cells), showed that at 24 h postimmunization there were 50% less CD69^+^CD62L^+^ CD4 T cells in mice that received both antigens compared to mice that received STm only (Fig. 5A). This suggested that although the total number of activated T cells eventually reached comparable levels, at the earliest stages of the response there was some defect in T‐cell priming, possibly at the stage when DCs and T cells interact.

To examine whether sFliC altered the ability of DCs to activate antigen‐specific T cells EYFP mice were immunized with STm to generate a pool of STm‐specific primed T cells. After 7 days splenic T cells were enriched and 10^7^ of these STm‐experienced, EYFP^+^ T cells were transferred into WT recipients to generate chimeras. Twenty‐four hours later these chimeras were immunized with PBS, STm, sFliC, or both antigens and 24 h afterwards, EYFP‐activated splenic CD4 T cells were assessed by flow cytometry. Mice that received both antigens had a significantly lower proportion and number of EYFP‐T cells expressing CD69 than mice that only received STm (Fig. 5B). Confocal microscopy confirmed the flow cytometry results and showed there were more CD69^+^ T cells in the T zone of STm‐infected mice than in mice that received other combinations of antigen (Fig. 5B lower panel). Collectively, these results suggest that sFliC coimmunization alters early T‐cell activation in a manner that is likely to be dependent upon the interaction of T cells with antigen‐presenting cells.

### Coimmunization with sFliC and STm alters the activation of DCs and their cytokine profile

**Figure 6:**
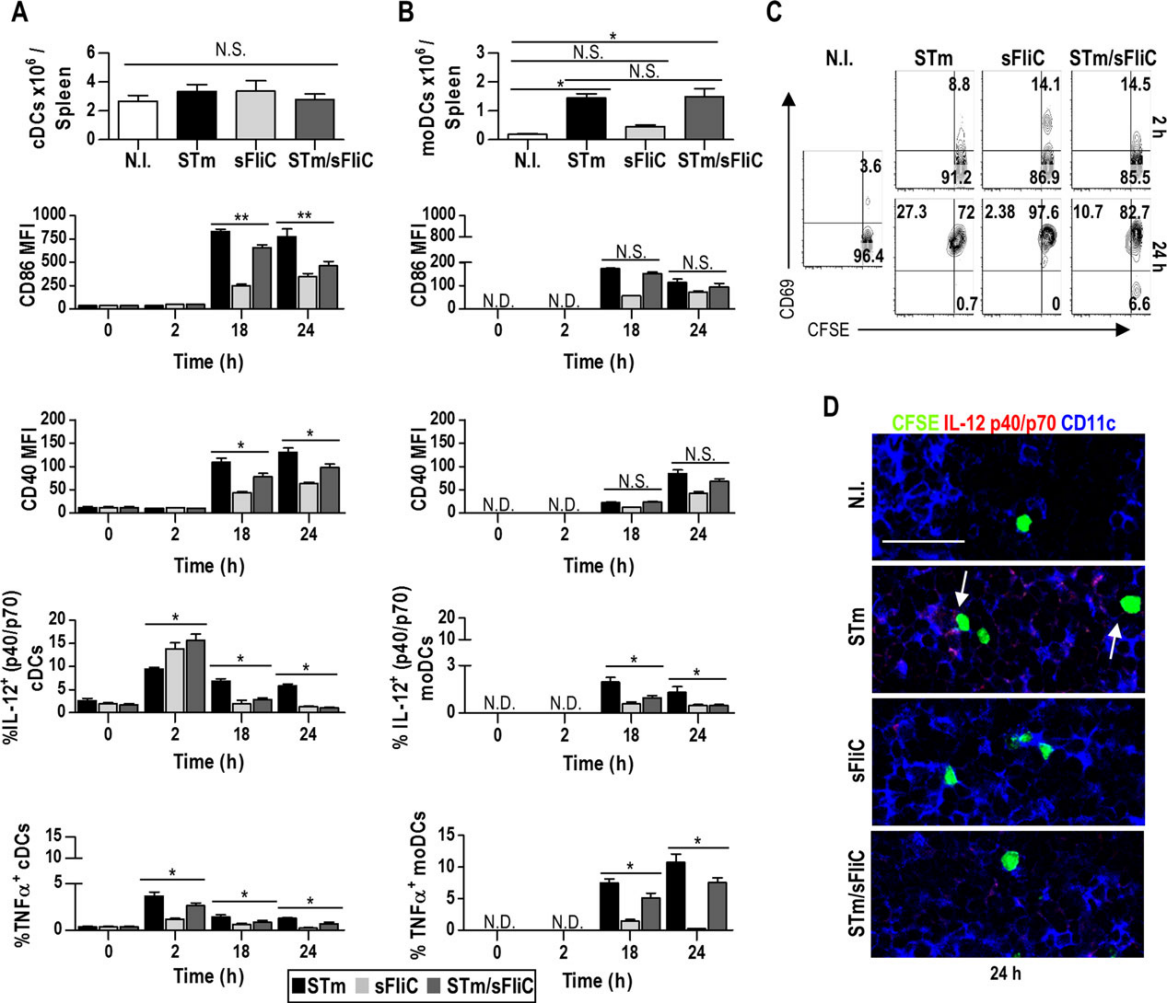
cDCs phenotype and cytokine profile are altered when sFliC is coimmunized with STm in comparison with the single immunizations. (A and B) WT mice were immunized with 5 × 10^5^ STm (black), 20 μg sFliC (light gray), or both (dark gray). Absolute number of (A) cDCs (Lin(B220/CD3/NK1.1)^−^CD64^−^ MHC‐II^+^CD11c^+^) and (B) moDCs (Lin(B220/CD3/NK1.1)^−^CD64^+^Ly6C^+^) 24 h postimmunization were calculated by FACS analysis. CD86 and CD40 expression 2, 18, and 24 h postimmunization was measured by FACS. Percentage of IL‐12 p40/p70 and TNFα 2, 18, and 24 h postimmunization was measured by FACS. Data are shown as mean ± SD (*n* = 3 mice/group) and are representative of three independent experiments. *p ≤ 0.01; **p ≤ 0.001, by 1 way ANOVA. (C) WT mice were transferred i.v. with 10 × 10^6^ CFSE labeled SM1 splenocytes, 24 h posttransfer recipients were nonimmunized or immunized as in (A and B). Representative contour plots show SM1 T‐cell (CFSE^+^CD3^+^CD4^+^) activation defined as CD69 expression 2 and 24 h postimmunization. Numbers indicated frequency within the quadrant. (D) Composite confocal representative images of recipients splenic T zones 24 h postimmunization, showing CFSE (green), CD11c (blue), and IL‐12 p40/p70 (red). (Scale bar: 25 μm). Arrows indicate close interaction between CFSE cells and CD11c^+^IL‐12 p40/p70^+^ cells. Images are representative of three independent experiments.

Since sFliC coimmunization altered priming and T‐cell polarization to Th1 it suggested that sFliC may modulate DCs, as these are the most efficient cell‐type for priming naïve T cells. The optimal induction of IFN‐γ in CD4 T cells after STm infection requires the presence of cDC and moDC 18. Each of these DC subsets has a specific cytokine signature, so that at 24 h post‐STm cDC are the predominant source of IL‐12 and moDC of TNFα. After infection with attenuated STm in susceptible strains of mice, clearance of bacteria is not dependent upon TNFα 23. We hypothesized that the defect in Th1 differentiation reflected a defective accumulation of moDCs. To examine this, the kinetics of the DC response after immunization with each antigen separately or together was assessed (gating strategy shown in Supporting Information Fig. 1C). As previously reported 18, at 24 h postimmunization numbers of cDCs were similar in nonimmunized mice or those immunized with STm or sFliC (Fig. 6A). There was an approximate tenfold increase in moDCs after STm infection, which the coimmunization with sFliC did not alter (Fig. 6B). This suggested that although numbers of DCs were similar there may be some defect in their function. Therefore the activation phenotype and cytokine profile of cDCs and moDCs was examined. Analysis of costimulatory molecules expression during the critical first 24 h window after immunization showed that coimmunized mice had significantly lower levels of CD86 and CD40 expression on cDC compared to STm alone (Fig. 6A; CD80 expression showed a similar profile as CD86, data not shown). Moreover, the kinetics of cytokine production by the DC populations after STm was substantially altered by the presence of sFliC. In cDCs at 2 h after sFliC, or sFliC and STm together there was a pronounced spike in IL‐12 p40/p70 expression, which was higher than in cDCs from STm‐only infected animals. This then fell rapidly by 18 h the proportion of cDCs expressing IL‐12 p40/p70 was below that of noninfected animals for all groups. Critically, numbers of cDCs producing IL‐12 p40/p70 at 18 and 24 h were significantly higher in the STm‐only infected than in the other two groups (Fig. 6A). This suggested that DCs may have become exhausted, probably due to the coordinated recognition of STm and sFliC. In contrast, in mice only receiving STm a modest proportion of moDCs expressed IL‐12 p40/p70, but substantially more expressed TNF‐α (Fig. 5B), reflecting earlier findings 18. Strikingly, in coimmunized mice there was a significant reduction in the proportion of moDCs expressing TNF‐α (Fig. 6B).

Although there was a much higher frequency of DCs producing Th1‐associated IL‐12 p40/p70 at 2 h in sFliC and STm/sFliC coimmunized mice, this is not likely to contribute to T‐cell priming by these DCs since there is no upregulation of the costimulatory molecules CD40 and CD86 at this time, and in CD86‐deficient mice T‐cell activation after STm or both antigens is absent (data not shown). That DCs have not primed T cells at this point was supported by the vast majority of SM1 T cells not expressing CD69at 2 h postimmunization, (Fig. 6C), when there is the spike in IL‐12 p40/p70 production (Fig. 6A) in the sFliC and the STm/sFliC‐treated groups. It is possible that there may be differences in the kinetics of activation, or other factors affecting T‐cell priming, that are not detected using these methods. In contrast, by 24 h the changes were dramatic with most of the T‐cell population expressing CD69and poised to begin dividing (Fig. 6C). Using this approach we also examined IL‐12 p40/p70 production in situ 24 h postimmunization, and showed that within the T zones of mice that only received STm some DCs were in close contact with SM1‐CFSE cells and producing IL‐12 p40/p70, confirming our flow cytometry data. This was not observed in the T zones of mice that received STm and sFliC (Fig. 6D). Therefore, co‐immunization with sFliC does not change the number of DCs recruited into the response, but modulates the activation of DC subsets and their expression of Th1‐associated cytokines at the time of priming.

**Figure 7:**
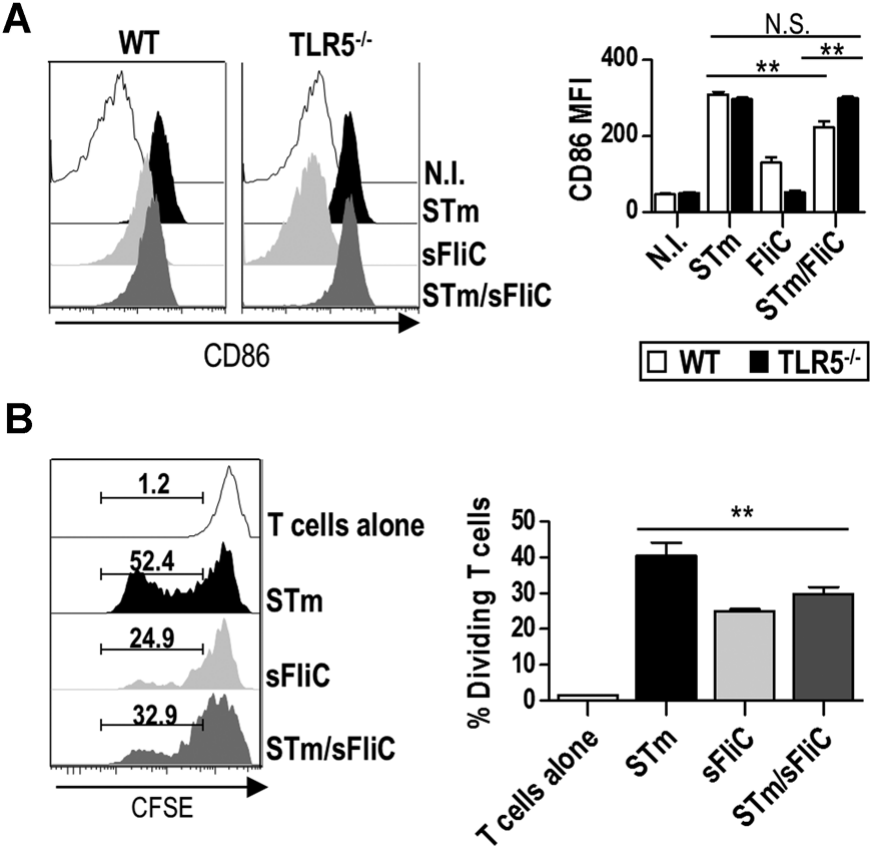
DCs conditioning in vivo mediates the T‐cell response. (A) DCs (Lin^−^(B220/CD3/NK1.1)MHC‐II^+^CD11c^+^) phenotype analysis of WT (white) and TLR5^−/−^ (black) mice 24 h postimmunization with 5 × 10^5^ STm (black), 20 μg sFliC (light gray), or both (dark gray). (Left) Representative histograms and (right) MFI CD86 values postimmunization are shown. (B) DCs were cell sorted from mice immunized as in (A) to use as APC (see detailed plots in Supporting Information Fig. 1C) 24 h postimmunization. T cells were cell sorted from 7 days STm‐infected (5 × 10^5^ SL3261) mice and CFSE labelled. Cells were cocultured in a 1:20 (DC:T) proportion for 4 days and T‐cell division was analyzed by CFSE dilution. (Left) Representative histograms and percentage of dividing T cells are shown. (A and B) Data are shown as mean ± SD (*n* = 3 mice/group) and are representative of three independent experiments. **p ≤ 0.001 by 1 way ANOVA.

To further study the effects observed by these antigens on DC maturation we examined the in vitro response of splenic cDCs purified from nonimmunized mice. Splenic DCs stimulated in vitro with STm, sFliC, or STm/sFliC for 1 h and cultured overnight showed no difference in the upregulation of CD86 and CD40 between STm and STm/sFliC. This may indicate that the observed defect in the upregulation of costimulatory molecules in the group treated with STm/sFliC was due to the interaction of different subsets activated in vivo or that moDCs, which are excluded from the in vitro system, contribute to these findings (Supporting Information Fig. 4). To determine whether the differences observed in DC function in the coimmunized mice were due to TLR5 stimulation we firstly examined CD86 expression in WT and TLR5^−/−^ mice 24 h postimmunization. TLR5^−/−^ mice coimmunized with STm and sFliC had a similar expression of CD86 to the STm‐only infected group, indicating that TLR5 was responsible for the observed differences in WT coimmunized mice (Fig. 7A). As expected we did not observe any upregulation of CD86 in TLR5^−/−^ mice immunized with sFliC alone.

Lastly, to confirm that the diminished T‐cell activation and polarization was due to defects in DC function, we performed ex vivo experiments using DCs from mice immunized with STm, sFliC, or both antigens to condition the DCs in vivo. DCs were FACS sorted, with a purity >97% (Supporting Information Fig. 1D). Sorted DCs were mixed with CFSE‐labeled CD4^+^CD62L^lo^ T cells sorted from mice infected with STm 7 days previously (at a ratio of 1 DC:20 T cells), again to increase the pool of antigen‐specific T cells to STm. To ensure all DCs had equivalent access to antigen, purified OMPS were added to the culture mix and the proportion of dividing T cells assessed 4 days later. DCs isolated from coimmunized mice were less capable than DC from STm‐immunized mice at driving T‐cell proliferation ex vivo (Fig. 7B). Therefore, coimmunization with sFliC and STm directly modulates DCs and their capacity to drive effective T‐cell activation.

## Discussion

While Th1 responses are essential for the control of many intracellular infections, they can also have undesirable effects, such as contributing to the immune‐mediated pathology often observed as infections are cleared 2. Therefore, understanding the factors that help limit the expansion of Th1 responses could be of significant therapeutic benefit. This study shows that the Th1 response to flagellated, but not aflagellate, STm can be selectively moderated after coimmunization with sFliC through a modulation of DC maturation and cytokine production. Despite the requirement for bacteria to be flagellated for the effects of sFliC coimmunization to be apparent, our data suggest it is not only impacting flagellin‐specific T cells. This is because the effects of sFliC coimmunization were observed after restimulation with both anti‐CD3 and purified OMPS and FliC‐specific T cells make only a small proportion of responding T cells in the early, endogenous response. Therefore, this offers potential as an intervention in reducing Th1‐mediated immunopathology during infection by TLR5‐stimulating bacteria. The reason why sFliC only affects the response to flagellated bacteria and not to aflagellated bacteria is unclear. A key point is that although the coimmunization only has effects in the presence of flagellated STm, the impact is on FliC‐specific and nonspecific T cells. This supports the concept that the initial influence is on DCs and their interaction with T cells and not necessarily on T cells. Since the effects of these interactions are still apparent at day 21 on both the clearance of infection and the numbers of IFN‐γ^+^ T cells, it demonstrates that the consequence of this coexposure to flagellated bacteria and sFliC has a lasting and dramatic effect on the host response. The context of antigen encounter in vivo is likely to be critical for this since in vitro experiments did not recapitulate the modifying effects seen in vivo. This suggests that DCs are likely to be influenced by their environment as well as their capacity to acquire antigen directly. While T‐bet expressing T‐cell numbers were dramatically reduced after coimmunization with sFliC, numbers of PD1^1ow^CXCR5^+^ T cells, associated with extrafollicular responses 22 were not and this reflected the normal early IgM and IgG antibody response to STm. In contrast cells with a phenotype shared with germinal‐center Tfh cells (PD1^+^CXCR5^+^) were not induced, despite being elevated when mice were immunized with sFliC alone. Collectively, this indicates that sFliC coimmunization can affect the development of the Th1 response to a broad repertoire of antigens within the bacterium yet is specific in only targeting flagellated bacteria. We focused on the response to STm in the spleen as it is a site where we have previously shown that after i.p. immunization with STm or sFliC, T‐cell priming occurs with similar kinetics. While systemic infection with STm, when given i.p. has rapid effects on homeostasis in sites such as the bone marrow 24 and thymus 25, we do not see T‐cell priming in sites such as the mesenteric LN or the popliteal lymph node until after the first day of immunization (unpublished observations). In contrast, sFliC administered i.p. can drive wide‐ranging responses in a number of secondary lymphoid tissues concurrently 26.

We initially used sFliC in these studies because it had previously been demonstrated to induce Th2 features to itself and coadministered antigens when administered as a soluble antigen. The capacity of Th2 antigens to alter Th1 responses have been demonstrated previously 27. Nevertheless, the effects described in this study do not appear to be related to the capacity of sFliC to induce Th2 features since IL‐4 production was absent after coimmunization. Furthermore, we have not observed these effects after coimmunization with other Th2 antigens. For instance after coinfection with STm and the Th2‐inducing helminth *N. brasiliensis* there is no abrogation in Th1 cell numbers or IFN‐γ production 10 nor after coimmunization with alum‐precipitated OVA and STm expressing OVA. Collectively, this indicates that the ability to moderate Th1 responses is probably unrelated to the Th2‐inducing properties of sFliC. The mechanism underlying the modulating properties of sFliC turned out to be surprising. In vivo and ex vivo experiments showed that DCs from coimmunized mice were poorer at activating T cells and inducing T‐cell proliferation than DCs from mice that only received STm. This indicates that after coimmunization sFliC acts on DCs. We observed two major effects on DCs after coimmunization, which were altered expression of the co‐stimulatory molecules CD40 and CD86 and an altered cytokine profile. The importance of effective induction of costimulatory molecules has been addressed extensively before and studies have shown that in the absence of optimal expression of costimulatory molecules by DCs then the functional quality of the T‐cell response is impaired 28, 29. Coupled with lower costimulatory molecule expression, there were fewer DCs expressing the Th1‐associated cytokines IL‐12 p40/p70 or TNFα by 18 h after immunization. This 12–18 h period is critical since this is when we have found that DCs prime T cells after systemic STm infection and so altered DC function is likely to lead to lower T‐bet induction 4, 6. Nevertheless, there may be events occurring before 18 h, associated with T‐cell priming that may not be detected by our technical approach. Potentially the C15.6 clone used to detect IL‐12 p40/p70 could also detect IL‐23 being produced from cDCs and future experiments will confirm whether cDCs produce only IL‐12 p40/p70 or if they also produce IL‐23.

How can the presence of sFliC have such effects? It is unlikely to be through simply inducing a tolerizing environment since these effects were observed in IL‐10‐deficient mice after coimmunization, and there was not a significant change in FoxP3^+^ T‐cell numbers and furthermore flagellin can act as an adjuvant to a number of coimmunized antigens such as OVA 16, 30. Data from multiple studies suggest that at the early time‐points examined here TLR5 expression is more critical on DCs and myeloid cells 31, 32, 33 than T cells. This is supported here since sFliC coimmunization in TLR5^−/−^ mice did not alter DC activation and there was no diminution in Th1 priming compared to WT mice. So, how would coimmunization affect the DC populations in vivo? One element to consider is that the response to several TLRs at once differs to when engaging just one. The potential synergy of TLR stimulation has been addressed previously, mainly by combining two ligands. However, after STm and sFliC there are more than two TLRs that can be ligated. In some systems it has been reported that the stimulation of TLR4 and TLR5 actually leads to an inhibition of IL‐12 p40/p70 and TNF‐α production 34. Other reports suggest that synergistic ligation of TLRs can enhance activation of transcription factors such as NF‐κB, but that the outcome can be variable depending on the time after stimulation and the particular TLR combination studied 35 and many of such studies have examined the response in vitro 34, 35, 36. A key implication of our results is that triggering DC responses through the additional exposure to sFliC can alter DCs in an unexpected and nonsynergistic manner. In this case the complex signals transduced after multiple TLR stimulation results in a lower level of DC activation and an impaired capacity to prime for Th1 cells. Therefore the interplay between TLRs is more nuanced than always enhancing a response. In our system, cells probably receive two tone signaling through TLR5, one via bacterial expression and one via encountering sFliC 37, 38. This means that it is likely that sFliC is only modulating the function of DCs that already contain bacteria. Additionally, previous data suggest that there is a division of labor between DCs for inducing Th1 cells and Tfh cells in this model of STm‐infection with moDCs being necessary for optimal priming of IFN‐γ‐secreting T cells but moDCs are dispensable for the generation of IgG2a‐switched extrafollicular plasma cells 18.

An implication of these findings is the capacity of STm and S. Typhi to modulate the expression of flagellin throughout the infection. It has been shown that Salmonella downregulates flagellin expression when it disseminates and the infection becomes systemic (spleen and liver) 39, 40, 41, 42, 43. Nevertheless, studies have shown that although flagellin production is reduced after systemic infection it is not necessarily switched off and can have additional activities as a monomer produced by intracellular bacteria 44. This suggests the activities described here could be relevant in mucosal and systemic sites and help modulate the response in target organs such as the liver, particularly as we have shown that parenteral administration of sFliC can impact the intestinal mucosa 26.

In summary, sFliC exposure can alter the tone of the adaptive response to flagellated bacteria. DCs are of central importance for this and therefore priming for Th1 responses requires a balancing of signals through engaging multiple TLRs 36, 45. This adds to the growing list of immunomodulatory activities that sFliC has on the host and provides additional focus for its translational potential in humans 46, 47.

## Material and methods

### Mice

Specific pathogen‐free 6–8 week C57BL/6 mice were purchased from Harlan Sprague–Dawley. SM1 [46], TCRβδ^−/−^, TLR5^−/−^, and EYFP mice were maintained in‐house. All animal procedures were carried out in strict accordance with local ethical approval from the University of Birmingham and the UK Home Office license (Project license 30/2850) as covered by the Animals (Scientific procedures) Act 1986.

### Antigen preparation and immunizations

sFliC was generated as previously described 19. Mice were immunized i.p. with 20 μg recombinant sFliC. STm SL3261 AroA^−^ 48 was cultured in Luria Bertani broth and harvested at mid‐log phase for immunization. Mice were infected i.p. with 5 × 10^5^ live STm in PBS. Aflagellated SL3261 (SW564) has a deletion of the fljB and fliC genes by replacement with an aminoglycoside phosphotransferase gene and chloramphenicol acetyltransferase cassette. In some experiments, the bacterial burden in tissues was evaluated by direct culturing.

### Cell preparation and FACS

Single cell suspensions from spleens and LNs were generated by mechanical disruption or collagenase IV (Worthington Biochemical) digestion (400 U/mL; 25 min; 37°C) when evaluating DCs. Cells were processed for FACS analysis accordingly with standard procedures. Antibodies are listed in Supporting Information Table 1.

For intracellular IFN‐γ and T‐bet staining, total splenocytes were plated at 6 × 10^6^ cells/mL with 1 μg/mL of anti‐CD28 Ab and restimulated in a precoated well with anti‐CD3 (10 μg/mL), OMPS (5 μg/mL), or culture medium for 6 h at 37°C, with BFA (10 μg/mL) for the last 2 h. Intracellular staining was performed by using the transcription factor staining buffer accordingly to manufacturer instructions (eBiociences).

When required, DCs were negatively enriched using MACS beads and LS columns (Miltenyi Biotec; CD19, CD5, and DX5 beads; purity ≥85%). Enriched DCs were plated at 3 × 10^6^ cells/mL and cultured overnight with GolgiStop^TM^ (8 μg/mL). Intracellular staining was performed using Cytofix‐Cytoperm (BD Biosciences). For in vitro cultures enriched DCs were incubated in antibiotic‐free medium with 5 × 10^3^ STm, 0.2 μg sFliC, or both for 1 h at 37°C. Cultures were then washed and resuspended in medium with antibiotics to culture overnight.

### ELISPOT analysis

For cytokine secretion the ELISPOT was perform as described before 18. In brief, IL‐4 ELISPOT was performed using a mouse IL‐4 ELISPOT kit (eBioscience, Hatfield, UK). A total of 4 × 10^5^ splenocytes were plated per well in medium alone or in presence of sFliC (5 μg/mL) and anti‐CD28 antibody (1 μg/mL) and cultured for 48 h at 37°C. B cell ELISPOT was performed as previously described 26. In brief, ELISPOT Plates (MultiScreen; Millipore) were precoated with 5 μg/mL of OMPS and 4 × 10^5^ splenocytes were plated per well. Cells were cultured for 6 h at 37°C. Spots were counted using the AID ELISPOT Reader System and AID software version 3.5 (Autoimmune Diagnostika).

### Generation of T‐cell chimeras and assessment of T‐cell priming

SM1 chimeras were generated as previously described 4 by transfer of 10^7^ CFSE‐labeled SM1 splenocytes i.v. into WT mice 24 h before immunization and the T‐cell‐specific response was evaluated at the indicated time‐points postimmunization. In some cases EYFP mice were infected i.p. with 5 × 10^5^ SL3261 to expand the antigen specific T‐cell pool. Spleens and LNs were harvested 7 days postinfection and T cells were positively enriched by MACS using CD5 beads (Miltenyi Biotech). Subsequently, 10^7^ enriched T cells were transferred i.v. into WT mice 24 h before immunization.

### Immunohistochemisty and confocal microscopy

Immunohistology was performed as described previously 19. Cryosections were incubated with primary unlabeled Abs for 45 min at RT before addition of either HRP‐conjugated or biotin‐conjugated secondary antibodies. Signal was detected using diaminobenzidine for HRP activity and naphthol AS‐MX phosphate with Fast Blue salt and levamisole for alkaline phosphatase activity.

Confocal was performed as previously described 18. For detection of cytokines in situ, sections were fixed with 4% paraformaldehyde for 20 min. Confocal images were acquired using a Zeiss LSM510 laser scanning confocal microscope with a Zeiss AxioVert 100 M. Signals obtained from lasers were scanned separately and stored in four nonoverlapping channels as pixel digital arrays of 2048 × 2048 (10X objective) or 1024 × 1024 (63X objective).

### OMPS‐specific ELISA

ELISA plates were coated with 5 μg/mL of OMPS (2 h at 4°C) and blocked with 1% BSA overnight at 4°C. Serum was diluted 1:100 in PBS/0.05% Tween and diluted stepwise; plates were incubated for 1 h at 37°C. Bound Abs were detected using alkaline phosphatase conjugated, goat anti‐mouse IgG, and IgG2a abs (Southern Biotech). Reaction was developed with Sigma‐Fast p‐nitrophenylphosphate (Sigma Aldrich). Relative reciprocal titers were calculated by measuring the dilution at which the serum reached a defined OD^405^.

### In vitro coculture of DCs and T cells

WT mice were immunized i.p. with STm (5 × 10^5^), sFliC (20 μg) or both for 24 h. Cell suspensions prepared as described above. Cells were preenriched by depleting CD19^+^, DX5^+^, and CD5^+^ cells by MACS beads before staining with CD11c, MHC‐II, and CD64 to FACS sort APCs. cDC (CD11c^hi^MHC‐II^high^CD64^−^) and moDC (CD11c^+^MHC‐II^+^CD64^+^) were sorted from STm and STm/sFliC immunized mice. In N.I. and sFliC‐immunized mice only cDCs were purified. Purity was ≥97% in all cases. T cells were isolated from 7 days STm‐infected WT mice infected. T cells were cell sorted (CD3^+^CD4^+^CD62L^low^) and CFSE labeled. DC were added in a 1:20 proportion (APC:T) and cultured for 4 days. Cells were harvested and analyzed by FACS to assess T‐cell division by CFSE dilution.

### Statistics

Statistics were calculated using the nonparametric 1 way ANOVA, Mann–Whitney sum of ranks test using Prism software with *p* values of ≤ 0.05 accepted as significant.

## Conflict of interest

The authors declare no financial or commercial conflict of interest.

**Abbreviations**
cDC: conventional DC
moDC: monocyte‐derived DC
OMPS: outer membrane proteins
sFliC: soluble flagellin
STm: *Salmonella* Typhimurium

## Supporting information

As a service to our authors and readers, this journal provides supporting information supplied by the authors. Such materials are peer reviewed and may be re‐organized for online delivery, but are not copy‐edited or typeset. Technical support issues arising from supporting information (other than missing files) should be addressed to the authors.

Figure 1. Gating strategies.
Figure 2. IFNγ modulation after sFliC/STm co‐immunization is not mediated by Treg or IL‐10 induction.
Figure 3. Flagellated and aflagellated STm strains induce similar infection.
Figure 4. In vitro stimulation of DCs with STm, sFliC or STm/sFliC reveals no difference.
Table 1. Antibodies and reagents used for FACS analysis immunohistochemistry, confocal, ELISPOT and ELISA.

Peer review correspondence
