# Supplementary material for: Soluble flagellin coimmunization attenuates Th1 priming to Salmonella and clearance by modulating dendritic cell activation and cytokine production
Source: Eur J Immunol. 2015 Jun 24;45(8):2299–311. doi: 10.1002/eji.201545564 (PMC4973836; doi:10.1002/eji.201545564)
Supplement: Supplementary file 1 — Figure 1. Gating strategies. Figure 2. IFNγ modulation after sFliC/STm co‐immunization is not mediated by Treg or IL‐10 induction. Figure 3. Flagellated and aflagellated STm strains induce similar infection. Figure 4. In vitro stimulation of DCs with STm, sFliC or STm/sFliC reveals no difference. Table 1. Antibodies and reagents used for FACS analysis immunohistochemistry, confocal, ELISPOT and ELISA. [file EJI-45-2299-s001.pdf]

Supporting Information

Figure 1. Gating strategies. (A) T-cell gating strategy. (B) Identification of GC B cells defined as $TCR^-CD138^-Fas^+GL7^+$. Plasma cells defined as $TCR^-B220^+CD138^+$. Tfh cells defined as $B220^-CD4^+CD62L^-CXCR5^+PD1^+$. (C) Identification of cDCs and moDCs on the basis of several markers. Cells were gated first for SSC and FSC as followed by pulse gate as shown in A. cDCs defined as Lin^-(CD3/B220/NK1.1)MHC-$II^{hi}CD64^-CD11c^{hi}$ in contrast moDCs were identified as Lin^-(CD3/B220/NK1.1)$Ly6C^{hi}CD64^+CD11c^+$MHC-II^+. (D) Pre and post-sorting plots showing the populations of cDCs and activated T cells used for the in vitro coculture assay.

Figure 2. IFNγ modulation after sFliC/STm co-immunization is not mediated by Treg or IL-10 induction. (A) WT mice were immunized with $5x10^5$ STm (black), 20 µg sFliC (light grey) or both (dark grey). Representative FACS plots show the expression of FoxP3 and CD25 on T cells ($CD3^+CD4^+$) 7 days post-immunization, numbers indicate percentage of the quadrant. (Right) Absolute number of $FoxP3^+CD25^+CD4^+$ T cells per spleen. (B) WT (white) or IL-$10^{-/-}$ (black) mice were non-immunized or immunized as in A, IFNγ response was evaluated 7 days post-immunization after in vitro restimulation with aCD3 by FACS. Data are shown as mean ±SD (n=4 mice/group) and are representative of four independent experiments.*$p \leq 0.01$, by 1 way ANOVA.

Figure 3. Flagellated and aflagellated STm strains induce similar infection. WT mice were non-immunized (white) or immunized with $5x10^5$ flagellated STm (black) or $5x10^5$ aflagellated STm (checked). (A) Numbers of cDCs and moDCs were evaluated 24 h post-immunization. At day 7 post-infection (B) absolute numbers of activated T cells ($CD62L^-CD44^+$ CD4 T cells) and (C) LPS IgM and IgG antibody response were evaluated. Data are shown as mean ±SD (n=4 mice/group) and are representative of two independent experiments.*$p \leq 0.01$, by 1 way ANOVA.

Figure 4. In vitro stimulation of DCs with STm, sFliC or STm/sFliC reveals no difference. Splenic DCs were isolated from WT mice and cultured for 1 h in the presence of medium (white), $5x10^3$ STm (black), 0.2 µg sFliC (light grey) or both (dark grey). Cultures were washed and resuspended in medium with antibiotics to culture

overnight. DC maturation was evaluated by the expression of CD86 and CD40 by FACS. Representative histograms are shown. Data are shown as mean ±SD (n=2-3 mice/group) and are representative of three independent experiments.*$p \leq 0.01$, by 1 way ANOVA.

Table 1. Antibodies and reagents used for FACS analysis immunohistochemistry, confocal, ELISPOT and ELISA.

Supporting information Figure 1
Flores-Langarica, et al

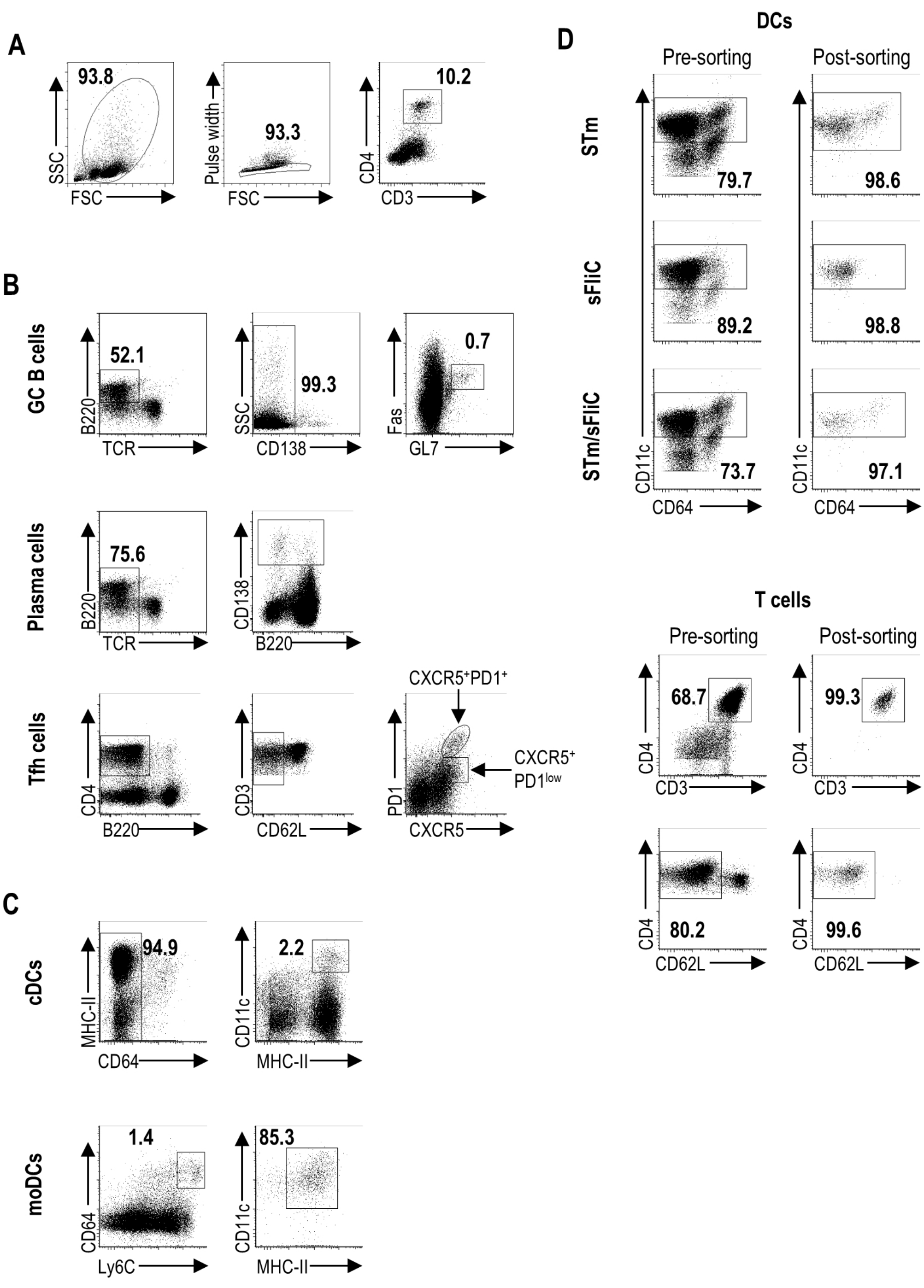

A

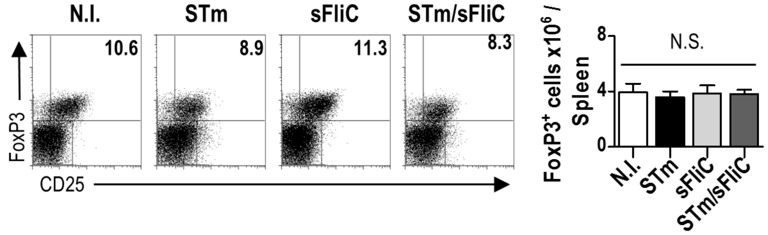
N.I.
STm
sFliC
STm/sFliC
10.6
8.9
11.3
8.3
FoxP3
CD25
FoxP3+ cells x10^6 / Spleen
N.S.

B

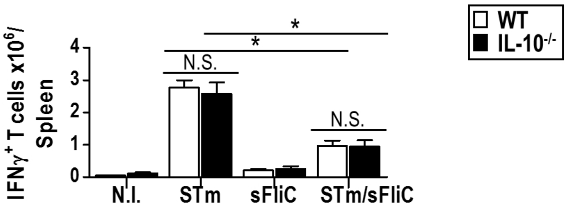
WT
IL-10-/-
IFNγ+ T cells x10^6/ Spleen
N.S.
*
*
N.S.
N.I.
STm
sFliC
STm/sFliC

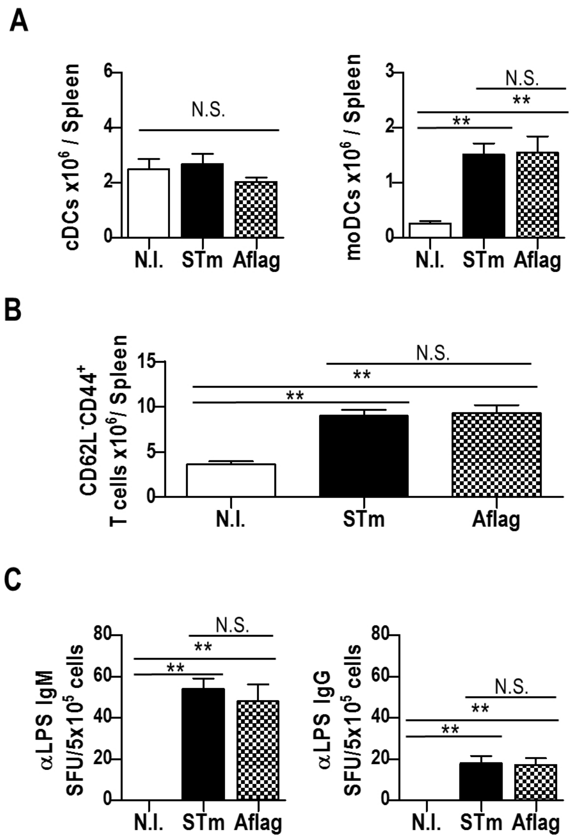
A
cDCs x10^6 / Spleen
N.S.
N.I.
STm
Aflag
moDCs x10^6 / Spleen
N.S.
**
**
B
CD62L$^-$CD44$^+$ T cells x10^6/ Spleen
N.S.
**
**
C
αLPS IgM SFU/5x10^5 cells
αLPS IgG SFU/5x10^5 cells

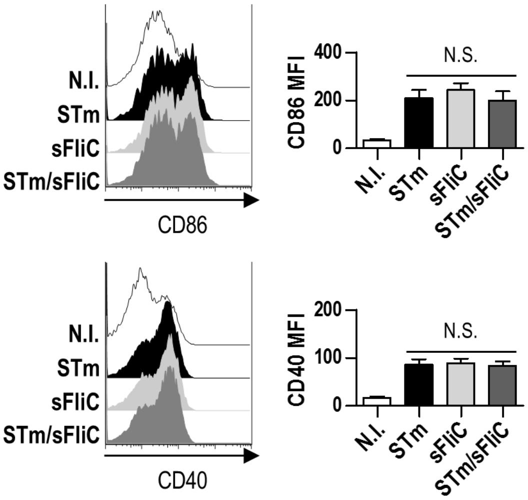
N.I.
STm
sFliC
STm/sFliC
CD86
CD86 MFI
400
200
0
N.S.
N.I.
STm
sFliC
STm/sFliC
N.I.
STm
sFliC
STm/sFliC
CD40
CD40 MFI
200
100
0
N.S.
N.I.
STm
sFliC
STm/sFliC

Reactivity	Isotype	Clone	Conjugate	Supplier
**B220**	Rat IgG2a, κ	RA3-6B2	FITC	BD Biosciences
**B220**	Rat IgG2a, κ	RA3-6B2	PO	Molecular probes
**CD3**	Armenian Hamster IgG	145-2C11	Purified	eBioscience
**CD3**	Armenian Hamster IgG	145-2C11	FITC	eBioscience
**CD4**	Rat IgG2b, κ	RM4-5	PerCP-Cy5.5	BD Biosciences
**CD4**	Rat IgG2b, κ	RM4-5	PB	BD Biosciences
**CD11b**	Rat IgG2b, κ	M1/70	PB	BD Biosciences
**CD11b**	Rat IgG2b, κ	M1/70	PE	BD Biosciences
**CD11c**	Armenian Hamster IgG 1, κ	N418	PE-Cy7	eBioscience
**CD40**	Rat IgG2b, κ	3/23	PE	BD Bioscience
**CD44**	Rat IgG2b, κ	IM7	PerCP-Cy5.5	eBioscience
**CD62L**	Rat IgG2a, κ	MEL-14	PE	BD Biosciences
**CD64**	Mouse IgG1, κ	X54-57.1	PE	Biolegend
**CD69**	Hamster IgG1 λ	H1.2F3	Purified	BD Biosciences
**CD69**	Hamster IgG1 λ	H1.2F3	APC	BD Biosciences
**CD86**	Rat IgG2a, κ	GL1	PE	BD Biosciences
**CD138**	Rat IgG2a, κ	281-2	APC	BD Biosciences
**CXCR5**	Rat IgG2a, κ	2G8	Purified	BD Biosciences
**Fas**	Armenian Hamster IgG2, λ	Jo2	PE-Cy7	BD Biosciences
**Foxp3**	Rat IgG2a, κ	FJK-16s	PE	eBioscience
**GL7**	Rat IgM	GL-7	PB	eBioscience
**IFNγ**	Rat IgG1	X.MG1.2	APC	BD Biosciences
**IgM**	Goat	Polyclonal	AMCA	Jackson Immunoresearch
**IgD**	Sheep	Polyclonal	Purified	Abcam
**IgM**	Goat	Polyclonal	AP	SouthernBiotech
**IgG**	Goat	Polyclonal	AP	SouthernBiotech
**IgG2a**	Goat	Polyclonal	AP	SouthernBiotech
**IL-12 p40/p70**	Rat IgG1	C15.6	PE	BD Biosciences
**Ly6C**	Rat IgG2c, κ	HK1.4	PerCP-Cy5.5	eBioscience
**MHC-II**	Rat IgG2b, κ	M5/114.15.2	APC	eBioscience
**NK1.1**	Mouse IgG2a, κ	PK136	FITC	BD Biosciences
**PD1**	Mouse IgG	J43	PE	eBioscience
**PNA**			Biotin	Vector

**T-bet**	Mouse IgG1, κ	4B10	PE Cy7	eBiosicence
**TCRαβ**	Armenian Hamster IgG	H57-597	PerCP-Cy5.5	eBiosicence
**TNFα**	Rat IgG1, κ	MP6-XT22	PE	eBioscience
**Armenian hamster**	Goat	Polyclonal	A647	Jackson Immunoresearch
**Mouse IgM**	Goat	Polyclonal	AP	SouthernBiotech
**Rat**	Rabbit	Polyclonal	Cy3	Jackson Immunoresearch
**Rat**	Rabbit	Polyclonal	Biotin	Dako
**Sheep**	Donkey	Polyclonal	POX	Binding Site

Supportive information Table 1. Antibodies and reagents used for FACS analysis immunohistochemistry and confocal.
