# Supplementary material for: Soluble flagellin coimmunization attenuates Th1 priming to Salmonella and clearance by modulating dendritic cell activation and cytokine production
Source: Eur J Immunol. 2015 Jun 24;45(8):2299–311. doi: 10.1002/eji.201545564 (PMC4973836; doi:10.1002/eji.201545564)
Supplement: Supplementary file 2 — Peer review correspondence [file EJI-45-2299-s002.pdf]

European Journal of Immunology

Soluble flagellin co-immunization attenuates Th1 priming to Salmonella and clearance by modulating dendritic cell activation and cytokine production

Adriana Flores-Langarica, Saeeda Bobat, Jennifer L Marshall, Juan Carlos Yam-Puc, Charlotte N Cook, Karine Serre, Robert A Kingsley, Leopoldo Flores-Romo, Satoshi Uematsu, Shizuo Akira, Ian R Henderson, Kai M Toellner and Adam F Cunningham

Correspondence: Dr. Adam Cunningham, University of Birmingham, Medical Research Council Centre for Immune Regulation, School of Immunity and Infection, Institute of Biomedical Research, B15 2TT, UK

Review Timeline:	Submission date:	06-Feb-2015
	First Editorial decision:	09-Mar-2015
	First Revisions received:	01-May-2015
	Accepted:	29-May-2015

Handling Executive Committee member: Prof. Caetano Reis e Sousa

Please note that the correspondence below does not include the standard editorial instructions regarding preparation and submission of revised manuscripts, only the scientific revisions requested and addressed.

First Editorial Decision -09-Mar-2015

Dear Dr. Cunningham,

Manuscript ID eji.201545564 entitled "Co-immunization with soluble flagellin attenuates Th1 priming to Salmonella through modulating dendritic cell activation and cytokine production" which you submitted to the European Journal of Immunology has been reviewed.

The comments of the referees are included at the bottom of this letter. A revised version of your manuscript that takes into account the comments of the referees will be reconsidered for publication.

You should also pay close attention to the editorial comments included below. **In particular, please edit your figure legends to follow Journal standards as outlined in the editorial comments. Failure to do this will result in delays in the re-review process.**

Please note that submitting a revision of your manuscript does not guarantee eventual acceptance, and that your revision will be re-reviewed by the referee(s) before a decision is rendered.

If the revision of the paper is expected to take more than three months, please inform the editorial office. Revisions taking longer than six months may be assessed by new referee(s) to ensure the relevance and timeliness of the data.

Once again, thank you for submitting your manuscript to European Journal of Immunology and we look forward to receiving your revision.

Yours sincerely,
Laura Soto Vazquez

On behalf of
Prof. Caetano Reis e Sousa

Editorial Office
European Journal of Immunology
e-mail: ejied@wiley.com
www.eji-journal.eu

****

Reviewer: 1

Comments to the Author

Flores-Langarica et al investigate the effect of co-immunization of soluble FliC and live S. typhimurium on the Th1 response to Salmonella. This is based on previous data showing that FliC administered in soluble form induces a TLR5-mediated Th2 response, which raises the question of whether sFliC can modulate the immune response to live Salmonella. Overall the study is well performed and the analyses are generally of good quality. The authors conclude that the mechanism underlying the modulating properties of sFliC are due to an influence on DC function rather than the Th2-inducing capacity of sFliC per se. However, the following concerns need to be addressed. Moreover, the biological significance of the data is unclear as there is no impact on bacterial clearance when examined at the same time point effects on the T cell

response are found.

In figure 6, what is the predicted biological effect of the difference in TNF production between the STM and STm/sFliC groups given there is no difference in splenic bacterial burden (Fig. 1D and 2)?

The authors use “IL-12” rather than indicating the subunit(s) detected (p40/p35/p70) when presenting and discussing data obtained using the antibody C15.6. The specificity of this antibody needs to be clearly stated. Is it possible that a good deal of what is detected is p40 homodimer as the heterodimer is in general difficult to detect, particularly in intracellular staining? Concluding that “IL-12” is detected may not be accurate as the p40 subunit is also a component of IL-23. Given that there is a Th-17 component to Salmonella immunity this needs to be considered and at least discussed in the manuscript. Thus, use of “IL-12” needs to be modified to address these concerns based on the specificity of the antibody used.

Figure 6. At 2 hrs post infection, cDC in mice given STm alone also had a spike in "IL-12" that was reduced at 18 and 24 hrs. On page 9, lines 7-12, the authors argue that this early spike of IL-12 in sFliC and STm/sFliC groups cannot contribute to T cell priming since they have not upregulated CD40 and CD86 at this time. However, the basal level of these molecules is already quite high so upregulation may have little impact on early T cell activation. Titrating in anti-CD86 antibodies in ex vivo experiments like in fig 7 or in in vitro experiments as suggested below would help address this. Also, the argument that in CD86-deficient mice T cells do not get activated presented in this section is not really relevant, as this is a complete absence of CD86 and cannot be compared to a high basal level whose further upregulation is differentially influenced by different stimuli. Likewise, that most SM1 T cells were CD69- 2 hrs post immunization also argues against a role in T cell priming may simply reflect a delay of kinetics relative to cytokine induction as indeed CD69 expression increases somewhere between 2 and 24 hrs post immunization and is not absent at 2 hrs (Fig 6C). The text in this section needs to be reworked to reflect these possibilities. Also, why was CD80 expression on DCs not shown? This should be shown given that CD80 versus CD86 engagement has been reported to influence T cell skewing.

p. 12 line 14 the authors state that normal DC activation occurred in TLR5-deficient mice and this is reflected in Figure 7A. However, it seems that the latter part of the sentence on lines 21-23 on p 9 of the manuscript is not consistent with this? Please clarify.

Fig. 7. Is there a direct and differential effect of sFliC, STm and sFliC/STm on DCs? That is, instead of priming the DCs in vivo where other cell types may influence the outcome of DC maturation in an indirect fashion, do (splenic) DCs exposed to sFliC, STm or both have different costimulatory molecule expression (CD80/86/40) or cytokine production (p40/p70) production and is this TLR5-dependent? This should be shown.

European Journal of Immunology

The finding that the modulating effect of sFliC requires that Salmonella express flagella is interesting. What possibilities do the authors have to explain this? This should be added to the manuscript. In the discussion, they speculate that sFliC is only modulating the function of DCs that already contain bacteria (p. 13 lines 1-2). This could be tested and would be an asset to the manuscript.

No impact on bacterial clearance was found at day 7, which is the time point the effects on the T cell response were found. Is there an effect of co-immunization of soluble FliC and bacteria on long-term survival or challenge to reinfection? This should be shown.

Is the data in Supportive information Figure 2 after restimulation? Is there a difference in frequency with anti-CD3 or OPMS as for IFNÎ³-producing cells?

Reviewer: 2

Comments to the Author

The work of Flores-Langarica et al. aims at characterizing the impact of flagellin on the immune response specific for a pathogen. This work shows co-administration of soluble flagellin (sFliC) with Salmonella enterica serovar Typhimurium by systemic route blunt the Th1 response. The authors also found that the inhibition of bacteria-induced Th1 response by flagellin is TLR5-dependent. They propose that this effect is driven by the reduced stimulation of dendritic cells (DC, both conventional and monocyte derived) when flagellin is administered to animals. The experiments are well carried out and presented.

The work is first a description of the biological effect of flagellin on bacterial immune responses, especially the CD4 T cell response. The second part attempts to define the mechanisms involved in the inhibition of Th1 responses. In between, there are also some data suggesting that this inhibitory effect of sFliC depends on the expression of flagellin by the live bacteria and is limited to T cell (since B cell response is not affected).

Major comments:
The authors only focused on the early effects on DC (within the first 24 hours) and adaptive immune responses at 7 days. They never address the dynamics of the adaptive responses and the consequence on the clearance of the bacteria. For instance, the authors could have provided data on adaptive responses at day 21 or later. Furthermore, their data at 7 days suggest that attenuation of Th1 response is not associated

with bacterial burden. Could they comment about these observations and provide data at later times?

The authors only studied the spleen whereas they use a systemic administration that is known to affect most lymphoid and non-lymphoid tissues, especially in response to sFliC. They should have discussed this latter issue.

The authors never analyzed the early outcome of pro-inflammatory response, particularly the production of blood cytokines including the antigen-presenting cells' cytokines that influence the CD4 T cell responses IL-12 (p40 and p70), IL-6, IL-1beta or the T cell-specific cytokines that can be produced very early after systemic injection of sFliC like IL-22, IL-17 and IFN-gamma. This issue is important to show that sFliC promotes these responses in the context of bacterial infection as it does alone. For example, one could expect sFliC mixed with bacteria is degraded by bacterial proteases (in particular when the authors used the flagellin-deficient strain). Such early-immediate responses may be crucial for an imprinting of the adaptive response, as it has been described for Leishmania infection.

sFliC did not impact on Th2 response but impacted on Th1 response. However, the bacterial burden in spleen was the same. Did the authors assess or Th17 cytokines?

It would have interesting to define whether the inhibition of bacteria-induced Th1 response by sFliC is a sequential process where sFliC and bacterial effect on DC can be unconjugated. sFliC is expected to diffuse very rapidly relative to bacteria and activate first DC in lymphoid and non-lymphoid tissue. Did the authors evaluate whether sFliC "tolerate" DC for response to bacteria

Minor comments:

OMPs are not defined.

Do the Salmonella injected by intraperitoneal route express flagellin (have flagella)?

The authors never claim about a role of inflammasome or crosstalk between TLR5 and inflammasome (either the NAIP/NLRC4 component or others). Inflammasome is really an main target when live invasive bacteria are used.

Figure 2: The authors did not explain what are the conditions for stimulation of T cells (anti-CD3, OMPs, other). If OMPs were used, can the authors provide data about expression of OMPs in aflagellated relative to flagellated bacteria?

Figure 2: Did the aflagellated and flagellated bacteria give rise to similar pathology? Did they induce infection of similar target cells? Do these bacteria stimulate similarly DC?

Figure 4: One would have expected to see circulating antibody titers specific for various antigens (at day 28 for example)

Figure 7: The authors should have presented IFN-gamma secretion in the in vitro presentation assay with CFSE labeled-cells. The authors have to change in the legend "cDCs" in "DCs"

<u>First Revision – authors' response- 1-May-2015</u>

Reviewer: 1

Comments to the Author

Flores-Langarica et al investigate the effect of co-immunization of soluble FliC and live S. typhimurium on the Th1 response to Salmonella. This is based on previous data showing that FliC administered in soluble form induces a TLR5-mediated Th2 response, which raises the question of whether sFliC can modulate the immune response to live Salmonella. Overall the study is well performed and the analyses are generally of good quality. The authors conclude that the mechanism underlying the modulating properties of sFliC are due to an influence on DC function rather than the Th2-inducing capacity of sFliC per se. However, the following concerns need to be addressed. Moreover, the biological significance of the data is unclear as there is no impact on bacterial clearance when examined at the same time point effects on the T cell response are found.

-The day 7 time-point was chosen because we knew the response to both STm and sFliC is well established at this time, although control of infection at this time is not dependent upon T cells [1].The intention of the work was to focus on the interaction between these responses at this early time and not widen it to be a study examining long-term control as this may dilute our central point that soluble flagellin (sFliC) can modulate the activities of DCs. Nevertheless, both reviewers raise the issue about what is the longer term effect of this interaction. To address this we have included new data in Fig. 3C that shows that co-immunized mice have lower numbers of IFNg-producing T cells and higher bacterial burdens at day 21 after infection, when CD4 T cells are important for bacterial clearance.

In figure 6, what is the predicted biological effect of the difference in TNF production between the STM and

European Journal of Immunology

STm/sFliC groups given there is no difference in splenic bacterial burden (Fig. 1D and 2)?

-Whilst TNFa plays an important role in controlling bacterial numbers in highly susceptible mice infected with a virulent strain of bacteria, in this less severe model of infection attenuating the TNFa response is not associated with altered control of bacterial numbers [2]. This is consistent with the findings presented here, and we have now added to the paper a sentence highlighting this point. In previous work we had shown that moDC and cDC collaborated in vivo to produce an optimal Th1 response and in vitro blocking TNFa from moDC alone could reduce this. We are unsure of the mechanism behind this although this is an area of interest for us, particularly as this model is now readily amenable to manipulation.

The authors use “IL-12” rather than indicating the subunit(s) detected (p40/p35/p70) when presenting and discussing data obtained using the antibody C15.6. The specificity of this antibody needs to be clearly stated. Is it possible that a good deal of what is detected is p40 homodimer as the heterodimer is in general difficult to detect, particularly in intracellular staining? Concluding that “IL-12” is detected may not be accurate as the p40 subunit is also a component of IL-23. Given that there is a Th-17 component to Salmonella immunity this needs to be considered and at least discussed in the manuscript. Thus, use of “IL-12” needs to be modified to address these concerns based on the specificity of the antibody used.

-The reviewer is correct that this clone could also pick up IL-23 and this has now been clarified throughout the manuscript.

Figure 6. At 2 hrs post infection, cDC in mice given STm alone also had a spike in "IL-12" that was reduced at 18 and 24 hrs. On page 9, lines 7-12, the authors argue that this early spike of IL-12 in sFliC and STm/sFliC groups cannot contribute to T cell priming since they have not upregulated CD40 and CD86 at this time. However, the basal level of these molecules is already quite high so upregulation may have little impact on early T cell activation. Titrating in anti-CD86 antibodies in ex vivo experiments like in fig 7 or in in vitro experiments as suggested below would help address this. Also, the argument that in CD86-deficient mice T cells do not get activated presented in this section is not really relevant, as this is a complete absence of CD86 and cannot be compared to a high basal level whose further upregulation is differentially influenced by different stimuli.

Likewise, that most SM1 T cells were CD69- 2 hrs post immunization also argues against a role in T cell priming may simply reflect a delay of kinetics relative to cytokine induction as indeed CD69 expression increases somewhere between 2 and 24 hrs post immunization and is not absent at 2 hrs (Fig 6C). The text in this section needs to be reworked to reflect these possibilities.

-In these experiments there was no change in the MFI of the cDCs of any co-stimulatory molecule in any of the immunized groups two hours after immunization and the median MFI was 10 for CD40 and 40 for CD86.

As would be expected there is some basal expression of these molecules on cDCs, and the levels found are high when compared with other APCs. This level of co-stimulatory molecule expression in steady-state mice is insufficient to start productive T cell responses as they induce T cells to become anergic [3]. None of the evidence at this time points to T cell priming being established, even in SM1 chimeras where the T cell precursor pool is enriched. When expression of these molecules increases just a few hours later, then this correlates with T cell activation. Therefore, we think the basal levels of co-stimulatory molecule expression do not contribute to T cell priming in this model and hope the reviewer can appreciate that there is likely to be little additional benefit of performing complex anti-CD86 titration experiments to test whether an unchanged expression of 40 confers an advantage over a total absence of CD86. In particular as none of the other data, eg a lack of CD69 upregulation in SM1 chimeras exposed to STm or STm/sFliC, supports T cell priming being established at this time. To address any potential concerns we have altered the wording in the results section and discussion to address the reviewer's concerns.

Also, why was CD80 expression on DCs not shown? This should be shown given that CD80 versus CD86 engagement has been reported to influence T cell skewing.

-CD80 expression was evaluated and showed a similar profile to CD86 expression. We considered that showing CD86 and CD40 offers a better profile of costimulatory molecules without being repetitive. To address the reviewer's concern the results section has been changed to state this:
"Analysis of co-stimulatory molecules expression during the critical first 24 h window after immunization showed that co-immunized mice had significantly lower levels of CD80, CD86 and CD40 expression on cDC compared to STm alone (Fig. 6A; and data not shown)."

p. 12 line 14 the authors state that normal DC activation occurred in TLR5-deficient mice and this is reflected in Figure 7A. However, it seems that the latter part of the sentence on lines 21-23 on p 9 of the manuscript is not consistent with this? Please clarify.

-We apologize about this lack of clarity. Those sections have been modified as follows. In the results the section now reads:
"To determine whether the differences observed in DC function in the co-immunized mice were due to TLR5 stimulation we firstly examined CD86 expression in WT and TLR5-/- mice 24 h post-immunization. TLR5-/- mice co-immunized with STm and sFliC had a similar expression of CD86 to the STm-only infected group, indicating that TLR5 was responsible for the observed differences in WT co-immunized mice (Fig. 7A)."

And the discussion:
"Data from multiple studies suggest that at the early time-points examined here TLR5 expression is more critical on DCs and myeloid cells [26-28] than T cells. This is supported here since sFliC co-immunization in

TLR5-deficient mice did not alter DC activation and there was no diminution in Th1 priming compared to WT mice."

Fig. 7. Is there a direct and differential effect of sFliC, STm and sFliC/STm on DCs? That is, instead of priming the DCs in vivo where other cell types may influence the outcome of DC maturation in an indirect fashion, do (splenic) DCs exposed to sFliC, STm or both have different costimulatory molecule expression (CD80/86/40) or cytokine production (p40/p70) production and is this TLR5-dependent? This should be shown.

-The reviewer raises an interesting point. We focus on in vivo studies as much as possible since we have found that the in vivo response often differs from the in vitro response. Two examples of this include the identification of the role of moDC in Th1 priming to STm and the role of CD103+ DCs in the mesenteric LN in the response to sFliC [4, 5]. The findings from these studies would not have been possible using in vitro studies alone. To address the reviewer's point we have now performed an in vitro study to examine the effects of these antigens on splenic DCs purified from naïve mice. These results showed that when splenic DCs are stimulated with STm, sFliC or both there is no difference in the level of CD86 and CD40 upregulation between the STm and the STm/sFliC groups. These results suggest that for these effects to be observed then the cells must be in their natural sites within the host. From these experiments we cannot conclude as to whether there is a direct or indirect of the antigens on the DCs that affects their behaviour in vivo as the process of taking the DCs from the host may have some modifying effects on their behaviour and of course ex vivo they do not receive signals from supporting cells such as from the stroma. An additional complication of this in vitro experiment is that the contribution of moDCs is excluded. An additional supportive information Figure 4 has been included showing these results. And an extra section has been included in the results:

"To further study the effects observed by these antigens on DC maturation we examined the in vitro response of splenic cDCs purified from non-immunized mice. Splenic DCs stimulated in vitro with STm, sFliC or STm/sFliC for 1 h and cultured overnight showed no difference in the upregulation of CD86 and CD40 between STm and STm/sFliC. This may indicate that the observed defect in the upregulation of costimulatory molecules in the group treated with STm/sFliC was due to the interaction of different subsets activated in vivo or that moDCs, which are excluded from the in vitro system contribute to these findings (Supplementary Information Figure 4)."

The finding that the modulating effect of sFliC requires that Salmonella express flagella is interesting. What possibilities do the authors have to explain this? This should be added to the manuscript. In the discussion, they speculate that sFliC is only modulating the function of DCs that already contain bacteria (p. 13 lines 1-2). This could be tested and would be an asset to the manuscript.

-We think the major interpretation of this is that only TLR5-expressing DCs that encounter flagellin in soluble form and in a bacterial-localised form are affected by this response. However, because these DCs are able to prime T cells of multiple specificities we think this explains why T cells of specificities other than to flagellin are affected. We are keen to address the reasons for this discrepancy but consider this a significant undertaking, since to do this it will involve a significant amount of cell sorting, signalling and ex vivo priming experiments. To address the reviewer's point we have expanded the discussion with some of these points.

No impact on bacterial clearance was found at day 7, which is the time point the effects on the T cell response were found. Is there an effect of co-immunization of soluble FliC and bacteria on long-term survival or challenge to reinfection? This should be shown.

-As discussed above we focused at day 7 to address the T cell response but have now included a day 21 time point, showing that the co-immunization of STm/sFliC does impact in the bacteria burden and that the defect on Th1 polarization is still observed. This data is included in Fig 3.

Is the data in Supportive information Figure 2 after restimulation? Is there a difference in frequency with anti-CD3 or OPMS as for IFNg-producing cells?

-We apologise for the lack of detail, the data presented in Supportive information Figure 2 is from an in vitro restimulation with anti-CD3. Since in previous experiments we observed similar results with anti-CD3 and OMPS we focused on anti-CD3. Changes have been made in the Results section and the figure legend has been changed to clarify this matter.

Reviewer: 2

Comments to the Author

The work of Flores-Langarica et al. aims at characterizing the impact of flagellin on the immune response specific for a pathogen. This work shows co-administration of soluble flagellin (sFliC) with Salmonella enterica serovar Typhimurium by systemic route blunt the Th1 response. The authors also found that the inhibition of bacteria-induced Th1 response by flagellin is TLR5-dependent. They propose that this effect is driven by the reduced stimulation of dendritic cells (DC, both conventional and monocyte derived) when flagellin is administered to animals. The experiments are well carried out and presented.

The work is first a description of the biological effect of flagellin on bacterial immune responses, especially the CD4 T cell response. The second part attempts to define the mechanisms involved in the inhibition of

Th1 responses. In between, there are also some data suggesting that this inhibitory effect of sFliC depends on the expression of flagellin by the live bacteria and is limited to T cell (since B cell response is not affected).

Major comments:
The authors only focused on the early effects on DC (within the first 24 hours) and adaptive immune responses at 7 days. They never address the dynamics of the adaptive responses and the consequence on the clearance of the bacteria. For instance, the authors could have provided data on adaptive responses at day 21 or later. Furthermore, their data at 7 days suggest that attenuation of Th1 response is not associated with bacterial burden. Could they comment about these observations and provide data at later times?

-We have addressed this point above as it was also raised by reviewer 1. Essentially we focused on times up to day 7 because we wished to examine the development of the response. An attractive reason to do this is to look at a time where bacterial numbers are similar in both groups and so issues such as persistence of antigen or other issues raised by differential bacterial colonization are not potential confounders. At day 7 control of this infection, in this model, is T-independent (from our group[1, 6] as well as others previously from other groups). At day 21, clearance is T-dependent. We have now shown in Fig 3C that at day 21 there is a difference in the rate of bacterial clearance, with the co-immunized group having higher bacterial burdens, and this group have fewer IFNg+ CD4 T cells.

The authors only studied the spleen whereas they use a systemic administration that is known to affect most lymphoid and non-lymphoid tissues, especially in response to sFliC. They should have discussed this latter issue.

-We focused on the spleen in this response because we find that T cell activation in this site occurs with similar kinetics to both STm and sFliC [1]. The reviewer is correct that sFliC disseminates rapidly through the host and that STm has effects on multiple sites concurrently. We have published on this by examining the response in the thymus and bone marrow and the effects on host immune homeostasis [6, 7]. We do not necessarily see the same level of synchronous T cell priming in multiple secondary lymph nodes after STm, for instance activated T cells and moDC are only found in the mesenteric lymph nodes days later than found in the spleen. In contrast, after sFliC there is concurrent DC activation and T cell priming in the spleen, mesenteric lymph nodes and popliteal, brachial and inguinal lymph nodes [5] (and unpublished). Therefore, we have restricted our assessments to the spleen in this study. We have added this section to the discussion to highlight this point.
“We focused on the response to STm in the spleen as it is a site where we have previously shown that after ip immunization with STm or sFliC, T cell priming occurs with similar kinetics. Whilst systemic infection with STm, when given ip, has rapid effects on homeostasis in sites such as the bone marrow[7] and thymus[6],

we do not see T cell priming in sites such as the mesenteric LN or the popliteal lymph node until after the first day of immunization (unpublished observations). In contrast, sFliC administered i.p. can drive wide-ranging responses in a number of secondary lymphoid tissues concurrently[5]."
The authors never analyzed the early outcome of pro-inflammatory response, particularly the production of blood cytokines including the antigen-presenting cells' cytokines that influence the CD4 T cell responses IL-12 (p40 and p70), IL-6, IL-1beta or the T cell-specific cytokines that can be produced very early after systemic injection of sFliC like IL-22, IL-17 and IFN-gamma. This issue is important to show that sFliC promotes these responses in the context of bacterial infection as it does alone. For example, one could expect sFliC mixed with bacteria is degraded by bacterial proteases (in particular when the authors used the flagellin-deficient strain). Such early-immediate responses may be crucial for an imprinting of the adaptive response, as it has been described for Leishmania infection.

-We have only looked in a limited manner at effects of immunization in the blood. This has been for a number of reasons. These are primarily based around having only detected small/negligible changes in blood cytokine levels (eg IFNg) after infection with attenuated STm and that when changes are detected then we are not sure where the cytokine was produced and by what cell type. For this reason we focus more on what is happening in the tissues. We think blood cytokine levels can be helpful diagnostically or prognostically but since there is probably some functioning of these in a concentration-dependent manner we have examined cytokines produced by the cells themselves. Therefore, we agree that linking findings in the blood to effects in the tissues is a worth topic to pursue, but we consider these to be part of a future study.

sFliC did not impact on Th2 response but impacted on Th1 response. However, the bacterial burden in spleen was the same. Did the authors assess or Th17 cytokines?

-We have not assessed IL-17 in the spleen in this study. In previous work[1] we found no evidence of IL-17 mRNA production from splenic T cells by RT-PCR and <1% of T cells producing this cytokine by restimulation, after sFliC immunization and so did not pursue this in the context of co-immunization in the current study.

It would have interesting to define whether the inhibition of bacteria-induced Th1 response by sFliC is a sequential process where sFliC and bacterial effect on DC can be unconjugated. sFliC is expected to diffuse very rapidly relative to bacteria and activate first DC in lymphoid and non-lymphoid tissue. Did the authors evaluate whether sFliC "tolerate" DC for response to bacteria

-The reviewer is correct regarding the likely rapid dissemination of sFliC. We do not think there is a tolerizing effect of this antigen on the response to STm and probably other antigens. The reasons for this include the

data in the paper showing a reduced Th1 response observed in IL10-deficient mice, the lack of change in Treg numbers (Fig), that this only occurred in the response to flagellated bacteria. In addition, we and others have shown that when there is co-immunization of flagellin with model antigens such as OVA [8-10] there is a strong response induced to these passenger antigens. For these reasons we do not think that flagellin necessarily is inducing a tolerizing effect. We have expanded the discussion to address this point:

“It is unlikely to be through simply inducing a tolerizing environment since these effects were observed in IL-10-deficient mice after co-immunization and neither was there a significant change in FoxP3+ T cell numbers and flagellin can act as an adjuvant to a number of co-immunized antigens such as OVA [16,29,30].”

Minor comments:

OMPs are not defined.

-OMPS are defined in Page 5 line 18: “This response was not only observed in anti-CD3 restimulated cultures but also when using purified STm outer membrane proteins (OMPS)…”

Do the Salmonella injected by intraperitoneal route express flagellin (have flagella)?

-We cannot be certain each individual bacterium expresses flagella. What we can say is that STm cultured the way described in the paper produce flagellin that is readily detectable by Western blotting of cell pellets, dominates the first 50 or so Salmonella protein hits by MALDI mass spectrometry in culture medium and drives rapid T cell priming in transgenic flagellin-specific T cells, priming which is absent in the absence of flagella.

The authors never claim about a role of inflammasome or crosstalk between TLR5 and inflammasome (either the NAIP/NLRC4 component or others). Inflammasome is really an main target when live invasive bacteria are used.

-The reviewer is correct that we do not make any claims on the inflammasome in relationship to this study; we simply do not have evidence either way. We do confirm a role for TLR5 in this process, but this may work in isolation or in conjunction with the inflammasome.

Figure 2: The authors did not explain what are the conditions for stimulation of T cells (anti-CD3, OMPs, other). If OMPs were used, can the authors provide data about expression of OMPs in aflagellated relative to flagellated bacteria?

-The data from Fig 2 were from in vitro cultures restimulated with anti-CD3. The figure legend has been changed to: "Intracellular IFNg in CD4+ T cells was evaluated 7 days post-immunization after in vitro restimulation with aCD3." OMPs will be similar between the groups as the predominant proteins are the porins OMPC, D and F and OMPA[11], which are present in the hundreds of thousands of molecules per cell and there will be the obvious exception of the presence of flagellin. We have not analysed the composition of OMPs in greater depth. We can say that flagellin, though present, is a minor species since the flagella are sheared off during the preparation process.

Figure 2: Did the aflagellated and flagellated bacteria give rise to similar pathology? Did they induce infection of similar target cells? Do these bacteria stimulate similarly DC?

-This is a great question. We did check that the infection induced by the aflagelleted bacteria was equivalent to that observed after flagellated bacteria. The response was similar in terms of numbers of DCs 24 hr post infection and both bacteria induced the accumulation of moDCs in the spleen similarly. T cell activation at day 7 was not significantly different either and neither was the IgM and IgG antibody response to LPS. All these data have been included in Supportive Information Figure 3. This of course does not mean there are not differences but that we did not detect them using the methods we applied.

Figure 4: One would have expected to see circulating antibody titres specific for various antigens (at day 28 for example)

-There are antibodies to STm at later time-points, we have previously published on the anti-FliC response after co-immunization at day 14[8]. This shows that there is an increase in anti-FliC IgG2a after co-immunization. We have now added the IgG and IgG2a antibody titres to OMPs at day 21. This shows no difference in this response at this time, this is added to Fig 4.

Figure 7: The authors should have presented IFN-gamma secretion in the in vitro presentation assay with CFSE labeled-cells. The authors have to change in the legend "cDCs" in "DCs"

-We did not assess this in vitro as we had assessed this extensively in vivo and used proliferation as a proxy. The figure legend has been changed as suggested.

References

1 Bobat, S., Flores-Langarica, A., Hitchcock, J., Marshall, J. L., Kingsley, R. A., Goodall, M., Gil-Cruz, C., Serre, K., Leyton, D. L., Letran, S. E., Gaspal, F., Chester, R., Chamberlain, J. L., Dougan, G., Lopez-Macias, C., Henderson, I. R., Alexander, J., MacLennan, I. C. and Cunningham, A. F., Soluble

flagellin, FliC, induces an Ag-specific Th2 response, yet promotes T-bet-regulated Th1 clearance of Salmonella typhimurium infection. European journal of immunology 2011. 41: 1606-1618.
2 Tite, J. P., Dougan, G. and Chatfield, S. N., The involvement of tumor necrosis factor in immunity to Salmonella infection. J Immunol 1991. 147: 3161-3164.
3 Bonifaz, L. C., Bonnyay, D. P., Charalambous, A., Darguste, D. I., Fujii, S., Soares, H., Brimnes, M. K., Moltedo, B., Moran, T. M. and Steinman, R. M., In vivo targeting of antigens to maturing dendritic cells via the DEC-205 receptor improves T cell vaccination. J Exp Med 2004. 199: 815-824.
4 Flores-Langarica, A., Marshall, J. L., Bobat, S., Mohr, E., Hitchcock, J., Ross, E. A., Coughlan, R. E., Khan, M., Van Rooijen, N., Henderson, I. R., Maclennan, I. C. and Cunningham, A. F., T-zone localized monocyte-derived dendritic cells promote Th1 priming to Salmonella. European journal of immunology 2011. 41: 2654-2665.
5 Flores-Langarica, A., Marshall, J. L., Hitchcock, J., Cook, C., Jobanputra, J., Bobat, S., Ross, E. A., Coughlan, R. E., Henderson, I. R., Uematsu, S., Akira, S. and Cunningham, A. F., Systemic flagellin immunization stimulates mucosal CD103+ dendritic cells and drives Foxp3+ regulatory T cell and IgA responses in the mesenteric lymph node. Journal of immunology 2012. 189: 5745-5754.
6 Ross, E. A., Coughlan, R. E., Flores-Langarica, A., Lax, S., Nicholson, J., Desanti, G. E., Marshall, J. L., Bobat, S., Hitchcock, J., White, A., Jenkinson, W. E., Khan, M., Henderson, I. R., Lavery, G. G., Buckley, C. D., Anderson, G. and Cunningham, A. F., Thymic function is maintained during Salmonella-induced atrophy and recovery. Journal of immunology 2012. 189: 4266-4274.
7 Ross, E. A., Flores-Langarica, A., Bobat, S., Coughlan, R. E., Marshall, J. L., Hitchcock, J. R., Cook, C. N., Carvalho-Gaspar, M. M., Mitchell, A. M., Clarke, M., Garcia, P., Cobbold, M., Mitchell, T. J., Henderson, I. R., Jones, N. D., Anderson, G., Buckley, C. D. and Cunningham, A. F., Resolving Salmonella infection reveals dynamic and persisting changes in murine bone marrow progenitor cell phenotype and function. Eur J Immunol 2014. 44: 2318-2330.
8 Cunningham, A. F., Khan, M., Ball, J., Toellner, K. M., Serre, K., Mohr, E. and MacLennan, I. C., Responses to the soluble flagellar protein FliC are Th2, while those to FliC on Salmonella are Th1. Eur J Immunol 2004. 34: 2986-2995.
9 Didierlaurent, A., Ferrero, I., Otten, L. A., Dubois, B., Reinhardt, M., Carlsen, H., Blomhoff, R., Akira, S., Kraehenbuhl, J. P. and Sirard, J. C., Flagellin promotes myeloid differentiation factor 88-dependent development of Th2-type response. J Immunol 2004. 172: 6922-6930.
10 McSorley, S. J., Ehst, B. D., Yu, Y. and Gewirtz, A. T., Bacterial flagellin is an effective adjuvant for CD4+ T cells in vivo. J Immunol 2002. 169: 3914-3919.
11 Nikaido, H., Molecular basis of bacterial outer membrane permeability revisited. Microbiol Mol Biol Rev 2003. 67: 593-656.

Second Editorial Decision - 26-May-2015

Dear Dr. Cunningham,

It is a pleasure to provisionally accept your manuscript entitled "Co-immunization with soluble flagellin attenuates Th1 priming to Salmonella and clearance through modulating dendritic cell activation and cytokine production" for publication in the European Journal of Immunology.

For final acceptance, please follow the instructions below and return the requested items as soon as possible as we cannot process your manuscript further until all items listed below are dealt with.

Please note that EJI articles are now published online a few days after final acceptance (see Accepted Articles: http://onlinelibrary.wiley.com/journal/10.1002/(ISSN)1521-4141/accepted). The files used for the Accepted Articles are the final files and information supplied by you in Manuscript Central. You should therefore check that all the information (including author names) is correct as changes will NOT be permitted until the proofs stage.

We look forward to hearing from you and thank you for submitting your manuscript to the European Journal of Immunology.

Yours sincerely,
Laura Soto Vazquez

on behalf of
Prof. Caetano Reis e Sousa

Editorial Office
European Journal of Immunology
e-mail: ejied@wiley.com
www.eji-journal.eu
